# Circumpolar distribution and carbon storage of thermokarst landscapes

**DOI:** 10.1038/ncomms13043

**Published:** 2016-10-11

**Authors:** D. Olefeldt, S. Goswami, G. Grosse, D. Hayes, G. Hugelius, P. Kuhry, A. D. McGuire, V. E. Romanovsky, A.B.K. Sannel, E.A.G. Schuur, M. R. Turetsky

**Affiliations:** 1 Department of Renewable Resources, University of Alberta, Edmonton, Alberta, Canada T6G 2H1; 2 Department of Integrative Biology, University of Guelph, Guelph, Ontario, Canada N1G 2W1; 3 Environmental Sciences Division, Oak Ridge National Laboratory, Oak Ridge, Tennessee 37831, USA; 4 National Remote Sensing Centre, Indian Space Research Organization, Balanagar, Hyderabad 500037, India; 5 Alfred Wegener Institute, Helmholtz Centre for Polar and Marine Research, Telegrafenberg A45, Potsdam 14473, Germany; 6 School of Forest Resources, University of Maine, Orono, Maine 04473, USA; 7 Department of Physical Geography, Stockholm University, Stockholm 106 91, Sweden; 8 U.S. Geological Survey, Alaska Cooperative Fish and Wildlife Research Unit, University of Alaska Fairbanks, Fairbanks, Alaska 99775, USA; 9 Geophysical Institute, University of Alaska Fairbanks, Fairbanks, Alaska 99775, USA; 10 Tyumen State Oil and Gas University, Tyumen, Tyument. Oblast 625000, Russia; 11 Center for Ecosystem Science and Society, Northern Arizona University, Flagstaff, Arizona 86011, USA

## Abstract

Thermokarst is the process whereby the thawing of ice-rich permafrost ground causes land subsidence, resulting in development of distinctive landforms. Accelerated thermokarst due to climate change will damage infrastructure, but also impact hydrology, ecology and biogeochemistry. Here, we present a circumpolar assessment of the distribution of thermokarst landscapes, defined as landscapes comprised of current thermokarst landforms and areas susceptible to future thermokarst development. At 3.6 × 10^6^ km^2^, thermokarst landscapes are estimated to cover ∼20% of the northern permafrost region, with approximately equal contributions from three landscape types where characteristic wetland, lake and hillslope thermokarst landforms occur. We estimate that approximately half of the below-ground organic carbon within the study region is stored in thermokarst landscapes. Our results highlight the importance of explicitly considering thermokarst when assessing impacts of climate change, including future landscape greenhouse gas emissions, and provide a means for assessing such impacts at the circumpolar scale.

## Abstract (web summary)

In thermokarst landscapes, permafrost thaw causes land subsidence with impacts on hydrology, ecology and biogeochemistry. Here, Olefeldt *et al*. produce circumpolar maps of thermokarst distribution, identifying that they cover 20% of the northern permafrost region, but store half the below-ground organic carbon.

## Graphical abstract

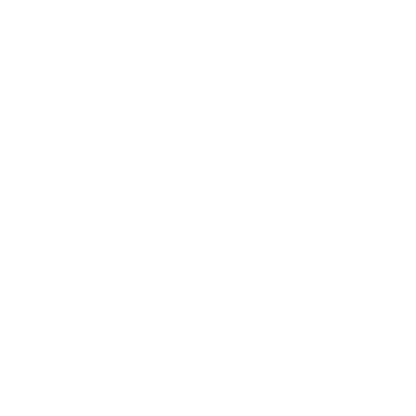

The northern circumpolar permafrost region stores ∼1,000 Pg soil organic carbon (SOC) in the upper 3 m^1^, similar in magnitude to the atmospheric carbon storage. Permafrost thaw due to climate change can occur both through widespread but gradual deepening of the seasonally thawed soil layer (that is, active layer deepening), and through the development of thermokarst landforms, which occur at discrete landscape locations and often affect the entire soil profile^2^,^3^. Thermokarst initiation at discrete locations occurs due to interactions of hydrology, soil properties, vegetation, geomorphology and disturbances, but fundamentally depends on the presence of excess ground ice that causes characteristic land surface subsidence when thawed^2^,^4^,^5^. Active layer deepening and the development of various thermokarst landforms each cause characteristic changes to soil environmental conditions and thus influence both the potential for SOC erosion and *in situ* rates of SOC mineralization into carbon dioxide (CO_2_) and methane (CH_4_). Current estimates indicate that greenhouse gas emissions from thawing permafrost soils will represent a major terrestrial biogeochemical feedback to climate change over this century, potentially on the same order of magnitude as global deforestation^3^. However, this permafrost carbon feedback remains poorly constrained, partly due to major uncertainties related to the role of rapid permafrost thaw through thermokarst^3^,^6^.

Thermokarst is generally not taken into account by the current generation of large-scale biogeochemical and earth system models for projecting future near-surface permafrost conditions^7^, SOC thaw^8^ and SOC mineralization into greenhouse gases^9^,^10^,^11^. This is despite a long history of field observations documenting thermokarst landform development at local scales^4^,^5^,^12^,^13^,^14^,^15^, and more recent local studies of post-thaw trajectories of carbon storage and greenhouse gas emissions^16^,^17^,^18^. The distribution of thermokarst landforms has been assessed at local and regional scales^19^,^20^,^21^,^22^,^23^, and a generalized map of permafrost hazard potential to human infrastructure is available at the circumpolar scale^24^. However, the inclusion of thermokarst in land surface and carbon cycle models has so far been in part hindered by the lack of a consistent circumpolar assessment of the distribution of thermokarst landscapes.

Here, we present a framework for using available spatial information on landscape characteristics within the northern boreal and tundra permafrost region to assess the distribution of thermokarst landscapes. We estimate that thermokarst landscapes cover 20% of the northern permafrost region and store up to half its SOC. By providing information on the distribution and carbon storage of different types of thermokarst landscapes, we aim to enable explicit consideration of the influence of thermokarst on future carbon cycling at the circumpolar scale.

## Results

### Assessing the distribution of thermokarst landscapes

**Figure 1:**
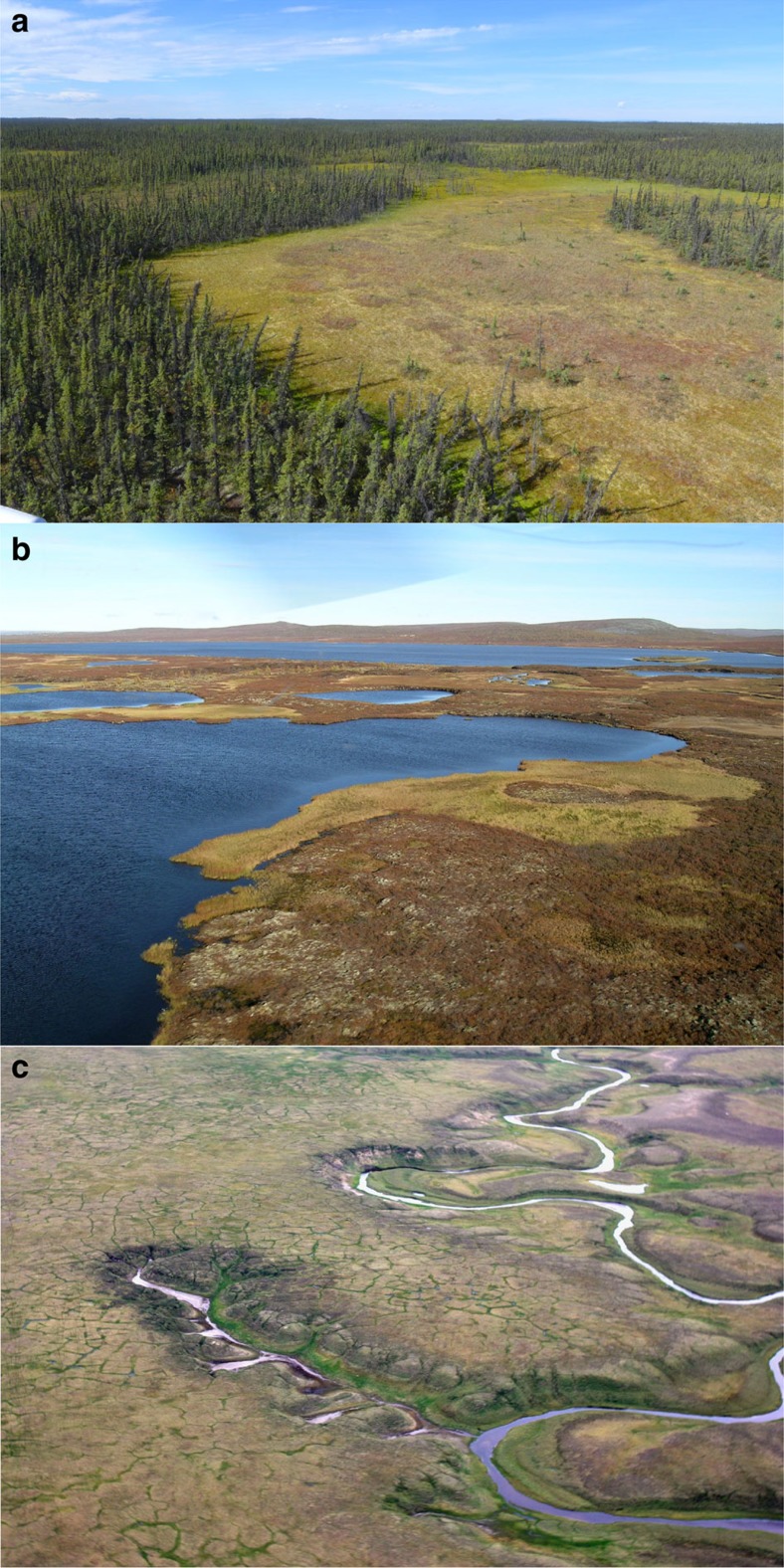
Examples of thermokarst landscapes. Photos of (**a**) wetland thermokarst landscape in the Northwest Territories of Canada, with characteristic thermokarst bogs (Photo: M. Helbig), (**b**) lake thermokarst landscape in northern Sweden with characteristic shallow thermokarst lakes (Photo: A.B.K. Sannel) and (**c**) hillslope thermokarst landscape on the Taymyr Peninsula, Russia, with characteristic thermal erosion gullies (Photo: G. Hugelius).

This study distinguishes between wetland, lake and hillslope thermokarst landscapes (Fig. 1). This distinction between different types of thermokarst landscapes is broad and it is recognized that a high degree of landscape diversity is retained within each type. Each thermokarst landscape type is defined by its association with a set of characteristic thermokarst landforms^5^ (see below). Characteristic thermokarst landforms of each thermokarst landscape preferentially co-occur spatially due to similarities in their dependencies on landscape characteristics for initiation and development. Nearly two dozen distinct thermokarst landforms have been identified in the permafrost affected northern boreal forest and tundra^4^,^5^. Our use of the term thermokarst landforms includes landforms traditionally termed thermo-erosional landforms. These differ from other thermokarst landforms in their dominance of lateral rather than vertical soil movement during landform development. We define the spatial extents of thermokarst landscapes to include both the areas of current thermokarst landforms and the areas susceptible to future thermokarst development. We further consider the three thermokarst landscape types to potentially overlap spatially, thus assuming that some landscape positions can be susceptible to the development of thermokarst landforms characteristic of more than one thermokarst landscape type. As an example, some landscape positions within tundra lowlands can potentially be susceptible to develop thermokarst landforms characteristic of any thermokarst landscape type.

**Figure 2:**
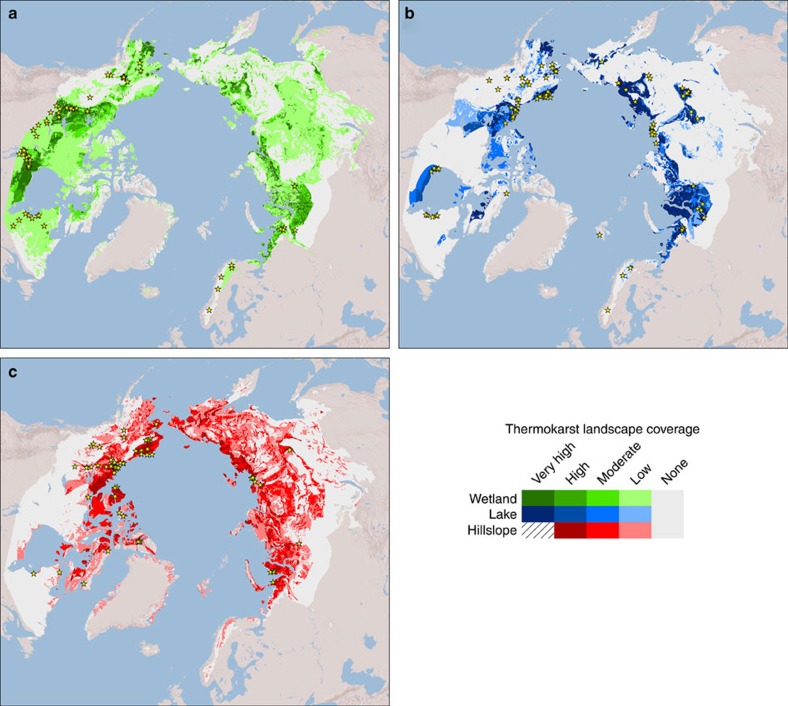
Distribution and regional coverage of thermokarst landscapes in the northern boreal and tundra circumpolar permafrost region. Differentiation is made for (**a**) wetland (green shading), (**b**) lake (blue shading) and (**c**) hillslope thermokarst landscapes (red shading). Coverage is classified as ‘Very High' (60–100% regional coverage), ‘High' (30–60%), ‘Moderate' (10–30%), ‘Low' (1–10%) and ‘None' (0–1%). Hillslope thermokarst landscapes are assumed to not reach ‘Very High' regional coverage. Yellow star symbols indicate study sites, described in literature, of thermokarst landforms characteristic of each thermokarst landscape (Supplementary Table 4-6). Background map of topography is based on GTOPO30 data (USGS, EROS, ESRI), accessed through ArcGIS 9.3.1.

**Figure 3:**
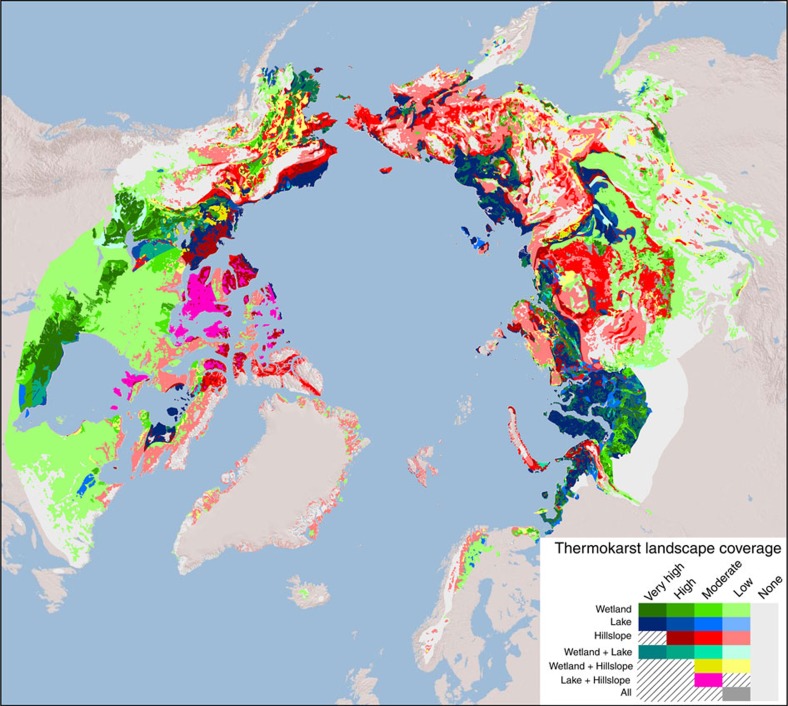
Dominant or co-dominant thermokarst landscapes within the northern boreal and tundra circumpolar permafrost region. The thermokarst landscapes with the greatest estimated regional coverage are shown. Co-dominance of two or all thermokarst landscapes is shown if estimated coverage classes are equal. Green, Blue and Red shadings indicate individual dominance of wetland, lake and hillslope thermokarst landscapes, respectively. Co-dominance is indicated by cyan (wetland and lake thermokarst landscapes), yellow (wetland and hillslope thermokarst landscapes), magenta (lake and hillslope thermokarst landscapes) and grey (equal coverage of all thermokarst landscapes). Dominant coverage is classified as ‘Very High' (60–100% regional coverage), ‘High' (30–60%), ‘Moderate' (10–30%), ‘Low' (1–10%) and ‘None' (0–1%). Coverage of hillslope thermokarst landscapes are assumed to not reach ‘Very High' regional coverage. Co-dominance combinations that have no representation in the map and are hashed in the legend. Background map of topography is based on GTOPO30 data (USGS, EROS, ESRI), accessed through ArcGIS 9.3.1.

Areal extents of thermokarst landscapes in this study are estimated through a conceptual modelling framework that weighs the perceived relative influence of landscape characteristics, including ground ice content^25^, sedimentary overburden thickness^25^, permafrost zonation^25^, terrestrial ecoregion^26^, topographical roughness^27^ and the presence of permafrost peat soils (histels)^28^ (Table 1). This study encompasses the boreal and tundra ecoregions^26^ within the northern circumpolar permafrost zones^25^, covering 12.4% of the world land area (Table 2). The spatial intersection of layers containing information on landscape characteristics used in the modelling framework yields >130,000 polygons, which we henceforth refer to as regions within the overall study area. Weights of landscape characteristics for determining regional coverage of thermokarst landscapes were decided through an expert elicitation, which included input from all co-authors as well as from members of the Permafrost Carbon Network^29^. This process was iterative, with consensus achieved through the sharing and discussion of experts' arguments for increasing or decreasing weights. The main arguments for the final weights are described in the paragraphs below. In the final model, regional coverage of thermokarst landscapes is binned into five classes; ‘Very High', ‘High', ‘Moderate', ‘Low' and ‘None'. Each coverage class corresponds to a fractional coverage of a region (Table 1). However, since we consider the three thermokarst landscapes to have the potential to spatially overlap within a region, the fractional regional coverage of individual thermokarst landscapes is adjusted to reflect this when more than one thermokarst landscape is present in a region (see ‘Methods' section for full explanation of how areal extent of thermokarst landscapes within a region is estimated from coverage classes). The resulting maps show the modelled regional coverage classes of wetland, lake and hillslope thermokarst landscapes, respectively (Fig. 2a–c), and the dominant or co-dominant thermokarst landscape types within each region (Fig. 3). Overall, we estimate that thermokarst landscapes cover 3.6 × 10^6^ km^2^, or ∼20% of the overall study area, with each of the three thermokarst landscapes contributing 5–8% each (Table 2).

### Wetland thermokarst landscapes

Typical thermokarst landforms in wetland thermokarst landscapes include thermokarst bogs, fens and shore fens, with development dependent on hydrological landscape position. Development causes transition from boreal forest or tundra dry shrub ecosystems into sedge or *Sphagnum* moss wetland ecosystems with near-surface water table position^4^,^5^,^15^. Thermokarst landforms are typically 0.5–10 ha but can reach sizes up to 100 ha. Development leads to 1–3 m land settlement but limited lateral soil movement.

The two landscape characteristics considered most important for estimating regional coverage of wetland thermokarst landscapes are histel soil coverage and topographic ruggedness (Table 1). We consider histels to be largely susceptible to the development of wetland thermokarst landforms due to their high-ground ice content^30^. Because histels have high-ground ice content, we did not further use landscape information on ground ice content by itself for estimating regional coverage (Table 1). Wetland thermokarst landscapes can dominate flat landscapes with extensive histels but are assumed to be largely confined to topographic lows in regions with more topographic ruggedness, including valley bottoms and adjacent to ponds and lakes^4^,^5^,^15^,^30^,^31^,^32^,^33^.

Secondary influences on regional coverage include permafrost zonation and sedimentary overburden thickness (Table 1). All else equal, we consider wetland thermokarst landscapes to have lower regional coverage in regions with thin sedimentary overburden and in colder permafrost zones. Thin sedimentary overburden is considered to limit the potential for vertical land subsidence and thus the development of characteristic thermokarst landforms. In colder permafrost zones, histels often occur in polygonal peatlands characterized by relatively thin organic soils^1^ and abundant ice wedges. In such polygonal peatlands it is more likely that thermokarst leads to the development of thermokarst troughs and pits develop^5^, which we consider characteristic of lake thermokarst landscapes (see below). In the non-continuous permafrost zones, our model allows wetland thermokarst landscape coverage to be greater than the permafrost coverage. This follows our definition of thermokarst landscapes, which includes both permafrost areas susceptible to future thermokarst development and non-permafrost areas of current thermokarst landforms^23^,^31^.

The resulting maps show ‘Very High' wetland thermokarst landscape coverage in well-known and extensive boreal peatland regions such as the West Siberian Lowlands, the Hudson Bay Lowlands and the Mackenzie River valley, but also indicate additional widespread ‘Low' coverage in much of boreal Canada and Russia (Fig. 2a). Wetland thermokarst landscapes in different settings will have distinct characteristics, for example, with thermokarst landforms developing from treed peat plateaus in boreal western Canada and Alaska^31^ or from non-treed palsas in tundra regions of Scandinavia and northwestern Russia^33^. In warmer permafrost zones it is also assumed that current thermokarst landforms dominate thermokarst landscapes, while areas susceptible to future thermokarst development are more prevalent in colder permafrost zones^15^.

### Lake thermokarst landscapes

Lake thermokarst landscapes are characterized by lake initiation, expansion, drainage and drainage basin development. Typical thermokarst landforms include deep, shallow and glacial thermokarst lakes, thermokarst lake basins, alas basins and thaw sinks^4^,^5^. Lake thermokarst landscapes are also considered associated with collapse pingos, and thermokarst troughs and pits, which all can have aquatic phases that may develop into thermokarst lakes^5^. Land settlement varies between 1 and 20 m, with potentially substantial lateral soil movement into inundated, anaerobic, conditions through wave action and colluvial processes. Resulting landforms vary greatly in size, with the largest landforms covering more than 5,000 ha.

Regional coverage of lake thermokarst landscapes is considered strongly influenced by topography, ground ice content, sedimentary overburden and permafrost zone^34^ (Table 1). Flat landscapes with thick sedimentary overburden and high-ground ice content are landscape characteristics considered important for abundant development of characteristic landforms^4^,^5^,^21^. Conceptually, we consider lake thermokarst landscapes to include the open-water areas of thermokarst landforms. Topography strongly limit the maximum attainable regional coverage of lake thermokarst landscapes in our model, and ‘Moderate' coverage is the maximum coverage possible in regions that do not have flat topography. Regional coverage of lake thermokarst landscapes is considered limited in warmer permafrost zones due to the lower permafrost coverage, but also due to the better drainage when thermokarst landforms develop in locations without underlying deeper permafrost layers^34^,^35^. Permafrost thaw in the boreal ecoregion is further considered less likely to lead to characteristic thermokarst landforms due to thicker surface organic mats that can reduce the mechanical removal of soil material^36^. Thermokarst lakes are however known to potentially occur in landscapes with thick organic soils if ground ice content is high^21^,^32^, and thus we do not consider histel coverage by itself to reduce the regional coverage of lake thermokarst landscapes (Table 1).

‘Very High' regional coverage of lake thermokarst landscapes is shown for several lowland tundra regions, including the Yukon delta, the Alaska north slope and the coastal regions along the Kara Sea, Laptev Sea and East Siberian Sea in Russia (Fig. 2b). These regions are known to have abundant thermokarst lakes^34^. ‘Very High' regional coverage further includes the boreal lowland along the Lena River and its tributaries, which is known to have a high abundance of alas landforms^14^. Lake thermokarst landscapes are further shown to overlap substantially with wetland thermokarst landscapes in major boreal peatland regions (Fig. 2a,b), albeit with lake thermokarst landscape generally having ‘Low' to ‘Moderate' coverage, while wetland thermokarst landscapes have ‘High' to ‘Very High' coverage.

### Hillslope thermokarst landscapes

Typical thermokarst landforms in hillslope thermokarst landscapes include active layer detachment slides, retrogressive thaw slumps, thermal erosion gullies, beaded streams and thermokarst water tracks^4^,^5^. Development can cause both substantial land subsidence and lateral soil transport through fluvial or colluvial processes. These thermokarst landforms are generally smaller than landforms of the other thermokarst landscapes, but can in some cases reach up to 10 ha. Hillslope thermokarst landforms are also to a greater degree limited in their development to landscape positions on moderate slopes or along watercourses, lake shores and coasts^4^,^5^,^22^. For that reason, we exclude hillslope thermokarst landscapes from attaining ‘Very High' regional coverage (Table 1). While hillslope thermokarst landscapes are considered most likely in undulating and hilly topography, ‘Moderate' regional coverage is still considered possible in flat regions due to the association of characteristic landforms with watercourses and shores^5^.

Primary factors, in addition to topography, considered to increase regional coverage of hillslope thermokarst landscapes are higher ground ice content, and colder permafrost zones^5^,^36^ (Table 1). Hillslope thermokarst development in warmer permafrost zones is considered limited since landscape positions preferential for characteristic thermokarst landforms is likely to already lack permafrost. Secondary influences considered for the regional coverage of hillslope thermokarst landscapes relate to the susceptibility of the landscape to erosion. Regions with thin sedimentary overburden or extensive histels, are thus less likely to be suitable for many of the characteristic thermo-erosional landforms typical of hillslope thermokarst landscapes^5^. Regions in the boreal ecozone are less likely to have extensive ice wedges that often act as points for hillslope thermokarst landform initiation^36^ (Table 1).

The resulting map shows ‘Moderate' and ‘High' regional coverage of hillslope thermokarst landscapes in many tundra regions, including the Alaska Seward Peninsula, the Alaska North Slope, the Mackenzie River Valley, the Canadian Arctic Archipelago and coastal regions along the Kara Sea, Laptev Sea and East Siberian Sea in Russia (Fig. 2c). This indicates that hillslope thermokarst landscapes often have their greatest concentrations in regions overlapping with ‘High' or ‘Very High' coverage of lake thermokarst landscapes (Fig. 2b). However, ‘Low' and ‘Moderate' regional coverage of hillslope thermokarst landscapes is widespread in vast continental regions with more pronounced topography in western North America, the Central Siberian Plateau and in the Russian Far East.

### Distribution of thermokarst landscapes

**Figure 4:**
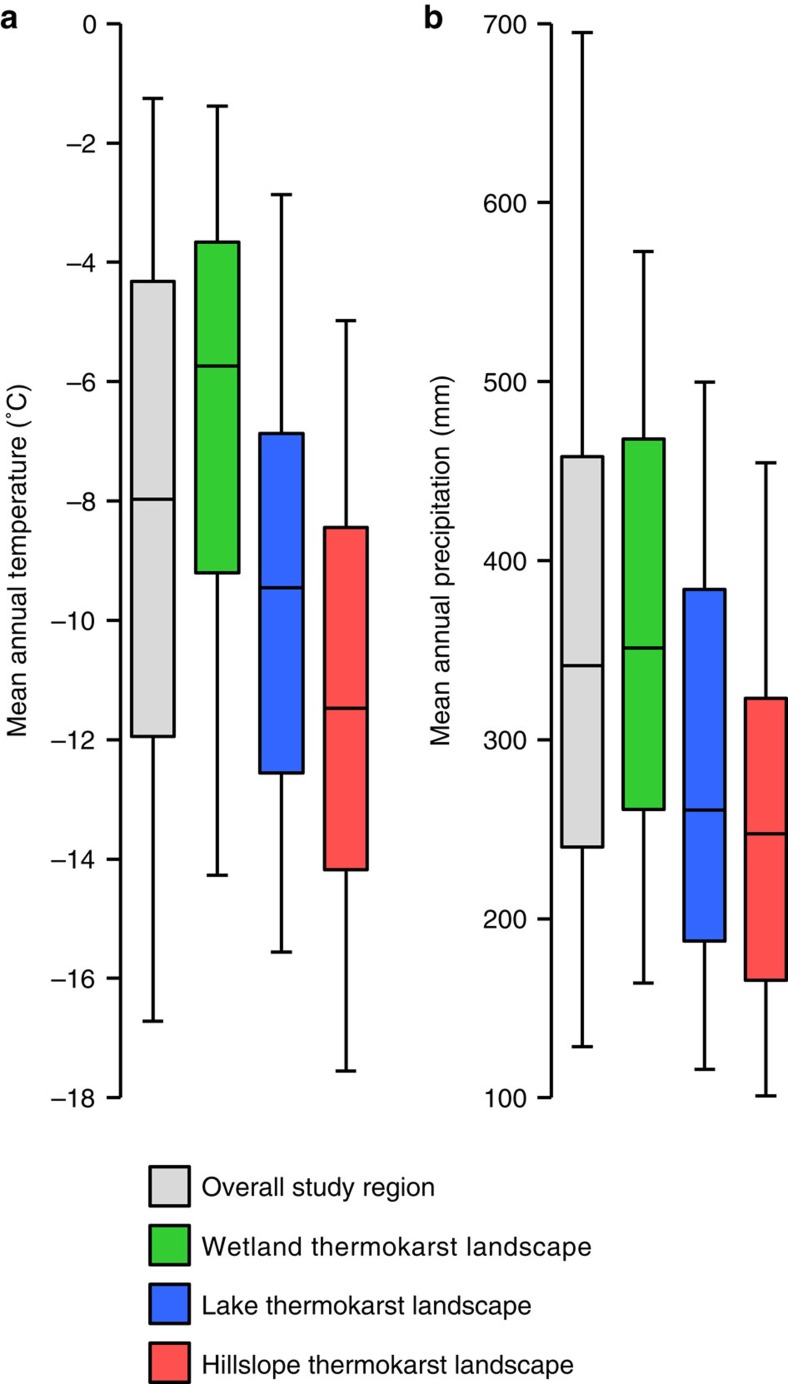
Distribution of thermokarst landscapes under current climate conditions. Data uses the area and climate for each region in the map of thermokarst landscape distribution. Mean annual (**a**) air temperature and (**b**) precipitation estimates for the period 1990–2010 is based on a fused CRU-NCEP re-analysis data set^31^. Grey boxes indicate the overall study region, while green, blue and red indicate wetland, lake and hillslope thermokarst landscapes, respectively. The boxes represent 25th and 75th percentiles (that is, indicating that 50% of the areas of the overall study region and of the three thermokarst landscape types fall within the box estimates). In addition, the bar indicates the 50th percentile, and whiskers showing 5th and 95th percentiles.

While the three thermokarst landscapes have similar estimated total areas (Table 2), their distributions differ with regards to both their spatial concentration and their relations to current climate conditions. Hillslope thermokarst landscapes are spatially least concentrated, with a majority (62%) of its total area found in regions with ‘Moderate' or ‘Low' coverage, compared with 32% and a mere 13% for wetland and lake thermokarst landscapes, respectively. Lake thermokarst landscapes are spatially the most concentrated, with 76% of its total area found in regions with ‘Very High' coverage. Thermokarst landscapes also differ in their relation to current climate conditions^37^, with hillslope thermokarst landscapes located in regions with colder and drier climate than lake thermokarst landscapes, which in turn are generally located in regions with colder and drier climate than wetland thermokarst landscapes (Fig. 4).

### Evaluation of mapped thermokarst landscapes

**Figure 5:**
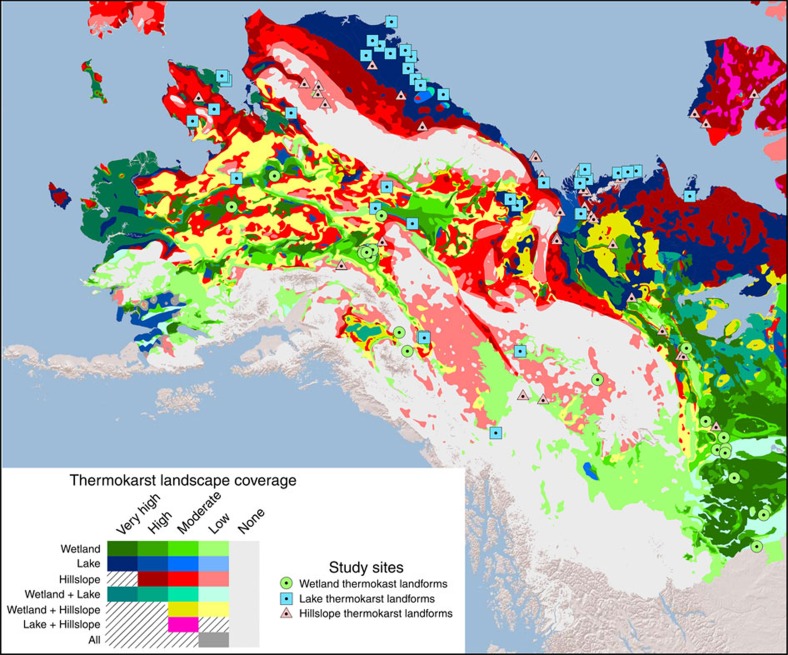
Locations of studied thermokarst landforms and mapped dominant thermokarst landscapes in Alaska and western Canada. Legend for thermokarst landscapes is same as in Fig. 3. Green circles, blue squares and red triangles represent studied thermokarst landforms characteristic of wetland, lake and hillslope thermokarst landscapes, respectively (Supplementary Tables 4–6). Background map of topography is based on GTOPO30 data (USGS, EROS, ESRI), accessed through ArcGIS 9.3.1.

Two independent approaches were used to evaluate the mapped regional coverages of thermokarst landscapes. First, we compiled a database of 225 locations of thermokarst landforms characteristic of wetland, lake and hillslope thermokarst landscapes (63, 92 and 69 landforms of wetland, lake, and hillslope thermokarst landscapes, respectively) described in 161 published studies (Supplementary Tables 4–6). The locations of these sites were compared with the corresponding mapped regional coverage of thermokarst landscapes (Figs 2a–c and 5). Roughly equal numbers of study sites (35–55 sites) were found within each coverage class. However, study site spatial concentrations were highest in regions with ‘Very High' coverage, reaching a concentration of 21.0 × 10^−6^ sites km^−2^, compared with 15.6, 8.0, 4.6 and 1.1 × 10^−6^ sites km^−2^ for ‘High', ‘Moderate', ‘Low' and ‘None' coverage, respectively. This pattern of decreasing study site concentrations in lesser coverage classes remained, with minor exceptions, when assessed for each thermokarst landscape separately (Supplementary Fig. 1). While study sites are not chosen randomly due to issues of accessibility in the north, increasing site concentrations in regions with greater mapped coverage of thermokarst landscapes is what would be expected from accurate maps.

The second approach for map evaluation is based on a comparison between the mapped regional coverages of thermokarst landscapes and an expert assessment of regional coverages based on satellite image interpretation and general site knowledge at 435 sites. Site locations for this expert assessment were chosen using a stratified random sampling approach, including 150 sites each for wetland and lake thermokarst landscape assessment, and 135 sites for hillslope thermokarst landscapes (Supplementary Fig. 2). Within each thermokarst landscape type, 50 sites were selected within regions of ‘None' coverage, and ∼25 sites within regions of each of the other coverage classes. Five experts per thermokarst landscape type independently assessed the coverage of thermokarst landscapes within a 20 km diameter circular polygon centred at each site location, using imagery in Google Earth (Google Inc., Mountainview, CA, USA, www.google.com/earth). All experts have previously published peer-reviewed articles on topics of relevance to the specific thermokarst landscape they assessed. Each expert was given instructions that included the definitions of the thermokarst landscapes, and of the five coverage classes used in the mapping framework. In their assessment of coverage at each site, experts were asked to take into account both their interpretation of satellite imagery available in Google Earth and their personal knowledge of local thermokarst conditions based, for example, on nearby field work. Experts were also asked to assess their confidence in their estimates of coverage at each site as high (satellite imagery highly suitable for accurate coverage estimate, and/or detailed personal knowledge of local thermokarst conditions), moderate (satellite imagery passable for accurate coverage estimate, and/or some personal knowledge of local thermokarst conditions) or low (satellite imagery unsuitable for accurate coverage estimate, and no personal knowledge of local thermokarst conditions). A consensus expert assessment of coverage for each site was determined based on the median coverage estimate of the five individual assessments.

**Figure 6:**
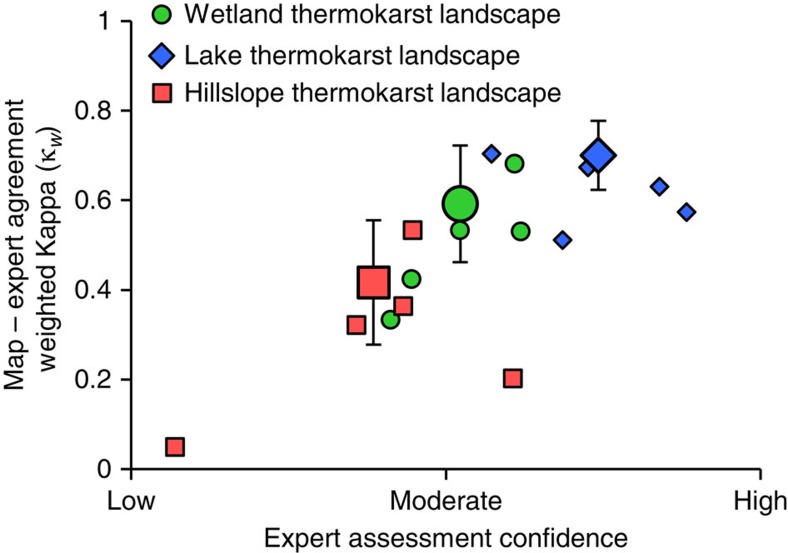
Relationship between expert confidence and the agreement between expert assessment and mapped coverages of thermokarst landscapes. Agreement between expert assessment and mapped coverages are estimated using a Kappa coefficient with quadratic weighting (κ_w_). Interpretation of κ_w_ has been proposed^33^, where 0.01–0.20 indicate slight agreement, 0.21–0.40 fair agreement, 0.41–0.60 moderate agreement, 0.61–0.80 substantial agreement and 0.81–1 almost perfect agreement. Small symbols represent average individual expert confidence and assessment agreement with mapped coverages, while the larger symbol for each thermokarst landscape type is based on the consensus expert assessment. Expert assessments of wetland, lake and hillslope thermokarst landscape coverages are indicated by green, blue and red symbols, respectively. Error bars indicate±2 s.e. of κ_w_, and is only shown for the consensus expert assessments.

The agreement between the expert assessment and the mapped coverages of thermokarst landscapes was evaluated through analysis of error matrices (Supplementary Tables 1–3; Fig. 3), including determination of a weighted Kappa coefficient^38^. The Kappa coefficient represents a measure of agreement between expert assessment and mapped coverage which takes into account the agreement that is expected due to chance alone. A Kappa coefficient of 1 indicates perfect agreement and a value ≤0 indicates no agreement^39^. A weighted Kappa coefficient further takes into account that not all disagreements are equally serious, as is the case of the ordered coverage classes in this study^40^. We chose to report a quadratic weighted Kappa coefficient, indicating that disagreements between expert assessment and maps are considered to be successively more egregious with greater divergence. Our map evaluation shows that experts who reported higher average confidence in their estimates of coverage also had greater agreement with the maps (Fig. 6). Both expert confidence and agreement further varied by thermokarst landscape type: the lowest confidence and agreement was found for hillslope thermokarst landscapes and the highest for lake thermokarst landscapes (Fig. 6). We note that expert level of confidence increases with the typical size of thermokarst landforms characteristic for each thermokarst landscape type, suggesting that the evaluation method is less suitable for assessing hillslope thermokarst landscape coverage due to issues with identifying typical landforms. Despite experts expressing overall limited confidence in their estimates of ground conditions, weighted Kappa coefficients for the consensus expert assessments of each thermokarst landscape type were 0.59±0.13 (±2 s.e.), 0.70±0.08 and 0.42±0.14, for wetland, lake and hillslope thermokarst landscapes, respectively (Fig. 6), indicating moderate to substantial agreement with the maps^39^,^40^.

### Carbon storage in thermokarst landscapes

Thermokarst landforms are often associated with landscapes that have high concentrations of below-ground organic carbon content^15^,^16^,^17^,^18^. To estimate the fraction of the below-ground organic carbon within the overall study region stored in thermokarst landscapes, we overlaid the resulting maps of thermokarst landscapes with estimates of regional 0–3 m SOC content^28^. The spatially interpolated data of regional overall SOC content further include information on constituent SOC concentrations (kg C m^−2^) for both permafrost soils (histels, orthels and turbels), non-permafrost soils (histosols, and a combined class of other mineral soil types), as well as on spatial coverage of non-soils (for example, rock lands) with negligible SOC concentrations. To estimate 0–3 m regional SOC storage of thermokarst landscapes, we first assumed that regional wetland thermokarst landscape SOC concentration could be approximated by using the regionally area-weighted SOC concentrations of histels and histosols (see ‘Methods' section). Using information on SOC concentrations from both permafrost and non-permafrost organic soils acknowledges that wetland thermokarst landscapes are a mosaic of non-permafrost thermokarst landforms and permafrost areas susceptible to future thermokarst. For lake and hillslope thermokarst landscapes, we then assumed that SOC concentrations were approximated by the area-weighted SOC concentrations of histels, orthels and turbels (that is, of permafrost soils). Lastly, regional SOC content of non-thermokarst landscapes was estimated as a residual by subtracting SOC content of thermokarst landscapes from the overall SOC content.

We estimate that thermokarst landscapes accordingly store ∼330 Pg SOC in the upper 3 m, constituting ∼30% of the total 0–3 m SOC storage within the overall study region (Table 2). Wetland thermokarst landscapes are estimated to have the highest 0–3 m SOC storage at ∼165 Pg SOC, about half of the total thermokarst landscape SOC storage, as a result of having the highest average SOC concentration at ∼115 kg C m^−2^. Both lake and hillslope thermokarst landscapes also had higher 0–3 m SOC concentrations at ∼80 and ∼75 kg C m^−2^, respectively, than non-thermokarst landscapes at ∼50 kg C m^−2^ (Table 2). While estimates of 0–3 m SOC storage in thermokarst sensitive landscapes include large uncertainties, stemming from both spatial extrapolation of SOC content from site specific pedon data^28^ and from our assumptions in determining coverage and SOC concentrations, our results support the notion that SOC within the overall study region is highly concentrated within thermokarst landscapes.

It is very likely that thermokarst landscapes would be found to store an even greater fraction of the total study region below-ground organic carbon storage if thermokarst lake sediments and SOC storage below 3 m depth were accounted for. Boreal peatlands within wetland thermokarst landscapes can reach depths well below 3 m^41^. Of great significance also are Yedoma landscapes and Arctic Ocean deltas, both dominated by thick frozen deposits that often are >20 m deep and thus have very large total organic carbon storage, despite low to moderate carbon content in the sediments^42^. Together they store an estimated ∼380 Pg C below 3 m in areas that spatially largely overlap with regions that have ‘Very High' coverage of lake thermokarst landscapes^1^,^18^,^42^. Including these Yedoma, lacustrine and deltaic sediments and their deeper carbon stores suggest that thermokarst landscapes store approximately half of the total study region below-ground organic carbon, despite covering only 20% of the area.

## Discussion

In this study we have described the development of maps indicating coverage of thermokarst landscapes, where the determination of regional coverage is tied to available information on landscape characteristics. While the accuracy of the resulting maps explicitly depends on a judicious consideration of the relative importance of different landscape characteristics, their accuracies are also critically dependent on the quality of the underlying data layers. While all data layers used in the assessment spanned the overall circumpolar study region, they varied greatly in their spatial resolution and level of detail. As such, the implications of combining data layers of varying resolution and quality need to be considered when interpreting and using the resulting maps. For example, the data layers with ground ice content, sedimentary overburden thickness and permafrost zonation have extremely broad regionalization^25^, and higher resolution assessments of these landscape characteristics at local scales show a heterogeneity that is not captured by the sources we used^20^,^42^. Several data layers, including those with permafrost zonation^25^ and histel distribution^28^, are further based on a compilation of different national inventories that each use somewhat different mapping approaches. This is likely to cause some inconsistencies in the resulting coverage estimates and levels of spatial detail for thermokarst landscapes among countries. Lastly, the overlay of the included data layers created more than 130,000 regions within the overall study region, thus generating a considerable fine-scale spatial detail (∼28% of polygons are <1 ha in size), which partially is caused by differences in data layer resolutions and chance polygon intersections rather than true differences in landscape characteristics. We therefore caution the interpretation of fine-scale patterns in the resulting maps, and acknowledge that more appropriate data layers may be available for assessing fine-scale patterns of thermokarst landscapes within specific regions^43^.

Evaluations of the resulting maps suggest, despite the inherent drawbacks of the mapping approach outlined above, that accuracy is adequate for many large-scale applications. Studied thermokarst landforms were disproportionally located within regions mapped with higher coverages of thermokarst landscapes, and expert site assessment of thermokarst landscape coverage had moderate to substantial agreement with mapped coverages. Neither approach, however, constitute a formal statistical analysis of map accuracy. For example, the agreement between expert assessment and mapped coverage of thermokarst landscapes cannot be directly interpreted to indicate map accuracy, since it is not known how well the expert assessments approximate ground truth conditions. While fine-scale pattern of thermokarst landscapes should be interpreted with caution, the resulting maps do correctly identify many of the larger regions known to have abundant thermokarst landforms, for example, landforms characteristic of wetland thermokarst landscapes in the Mackenzie River valley^15^, lake thermokarst landscapes in coastal lowland regions in eastern Siberia^42^ and Alaska^21^, and hillslope thermokarst landscapes in the foothills of the Brooks Range in Alaska^22^. Improved accuracy and level of detail for data layers of landscape characteristics, perhaps especially of ground ice conditions, would likely further improve the accuracy of the thermokarst map but would require a substantial coordinated effort of experts. Living databases with type and location of thermokarst landforms compiled by both members of the scientific community and the public could be an attainable and powerful way to further improve the resulting thermokarst maps, and would have many further uses. Overall we conclude, even with limitations, that the resulting maps represent an important advancement for the ability to assess broad scale impacts of accelerated thermokarst, for example, on infrastructure, landscape ecology, surface water quality, catchment hydrology, soil carbon cycling and greenhouse gas exchange with the atmosphere.

Vulnerability to thermokarst development is likely to increase this century both due to climate change and associated higher frequencies of disturbances such as wildfire and floods^2^,^44^,^45^. Gradual decadal increases in average annual temperatures have already been linked to an altered balance between thermokarst lake expansion and drainage^21^,^34^, and to accelerated expansion of thermokarst bogs^23^,^31^. Conversely, thermokarst landforms typical of hillslope thermokarst landscapes have been shown to have initiations and continued development largely confined to brief periods of extreme weather, particularly when unusually warm temperatures and high precipitation coincide^22^,^46^. Thresholds with regards to climate variables for thermokarst development thus likely depend on both the thermokarst landform considered, along with local ecosystem characteristics and hydrological landscape positions^2^. At broader scales it is however expected that it is the regional magnitude of climate change and associated shifts in disturbance frequency, rather than site specific thresholds, that will determine rates of future thermokarst initiation and development. Projections of climate change are largely consistent among climate models and forcing scenarios with regards to which regions that are likely to experience faster or slower than average climate change over this century^47^,^48^. The fastest change, with regards to both increasing mean annual air temperatures and precipitation, is generally expected for the coastal regions along the Arctic Ocean, likely driven by factors such as polar amplification, sea-ice retreat, and altered circulation patterns of oceans and atmosphere. Hence, regions with high coverage of lake and hillslope thermokarst landscapes in tundra regions in Eurasia, and in the northern Mackenzie River valley, are likely to be among the most vulnerable to accelerated thermokarst development. Noteworthy is that many highly vulnerable regions in Russia remain largely undescribed in English-language scientific literature (Fig. 2a–c), and thus may still hold surprises to our understanding of impacts of accelerated thermokarst, for example, on carbon cycling^49^.

We estimate that approximately half of the below-ground organic carbon within the study region is stored in thermokarst landscapes. Accurately accounting for the impact of thermokarst on C cycling is important since field evidence indicate that current models of high-latitude greenhouse gas exchange with the atmosphere may underestimate the impact of climate change by not explicitly considering thermokarst. Early stages of thermokarst development of specific thermokarst landforms is known to have the potential to cause large methane emissions to the atmosphere^50^,^51^ or downstream export of particulate and dissolved organic carbon into downstream ecosystems^52^,^53^. Rapid C loss in many thermokarst landforms is also likely facilitated by increased microbial access to SOC, as SOC thawed through thermokarst processes often remains unfrozen year round^17^,^34^. In addition, many regions indicated to have ‘Very High' coverage of lake thermokarst landscapes are dominated by Yedoma ground^42^, which has been found to contain SOC that is particularly microbially labile as a result of its unique genesis^54^,^55^. Despite the significant and rapid C mineralization following thermokarst, new accumulation of organic carbon in the form of peat or lake sediments could dominate over early losses on century to millscales^18^,^30^,^56^,^57^. These long term trajectories of post-thaw carbon cycling are important to consider since a large fraction of the SOC in thermokarst landscapes already has undergone one or more cycles of thermokarst development and permafrost re-aggradation. While our understanding of post-thaw trajectories of carbon cycling in thermokarst landforms is progressing^58^, new knowledge is needed with respect to how trajectories vary depending on landform type, SOC storage, SOC quality and landform history.

The permafrost carbon feedback, whereby climate change causes SOC mineralization and greenhouse gas emissions from permafrost soils to the atmosphere, is currently poorly constrained^3^,^9^,^10^,^11^. Combining maps of thermokarst landscapes with projections of future climates and emerging knowledge of post-thaw carbon cycling trajectories in thermokarst landforms, represents an essential step forward for estimating potential rates of permafrost SOC mineralization resulting from thermokarst. As such, the thermokarst landscapes maps produced in this study are important for reducing the uncertainty of the magnitude of the permafrost carbon feedback, and for enabling a comparison of permafrost SOC mineralization resulting from active layer deepening and thermokarst.

## Methods

### Modelling thermokarst landscape distribution

The spatial modelling framework implemented for estimating regional coverage of thermokarst landscapes in this study is based on an expert evaluation of the relative importance of a number of landscape characteristics. For this purpose, we used a set of six spatial circumpolar data layers describing key landscape characteristics; permafrost zonation (isolated, sporadic, discontinuous or continuous)^25^, ground ice content (<10%, 10–20% or >20%)^25^, sedimentary overburden thickness (thin or thick)^25^, terrestrial ecoregion (boreal or tundra)^26^, topographic ruggedness (flat, undulating, hilly or mountainous/rugged)^27^ and landscape coverage of histels (<10%, 10–30% or >30%)^28^. The spatial intersection of these layers results in 132,089 polygons (that is, regions) of variable size, with 28% of regions <1 ha and 13% >1,000 ha. The largest region covers 0.3% of the overall study region. Large lakes and glaciers were excluded from the overall study region and not part of the analysis.

The framework uses a subtractive score structure, with a unique set of scores for each thermokarst landscape type (Table 1). Points are subtracted from a maximum score for landscape characteristics considered to render a region less likely to have extensive coverage of thermokarst landscapes. The resulting score is categorized into five thermokarst landscape coverage classes, ranging from ‘None' to ‘Very High'. Each coverage class corresponds to an estimated fractional coverage of a region, with both a range and a mode for each class (Table 1). The mode fractional coverage is used to estimate areas of thermokarst landscapes within individual regions. Hillslope thermokarst landscapes were deemed improbable to exceed 60% regional coverage and thus the framework prevents it from attaining ‘Very High' coverage.

When two or all thermokarst landscapes are present in an individual region (that is, >‘None' coverage), then their fractional coverages are adjusted to take into account that we consider thermokarst landscapes to potentially overlap and occupy the same area. In this case, the thermokarst landscape type with the highest coverage class determines the cumulative fractional coverage of all three thermokarst landscapes. Adjusted fractional coverages for each thermokarst landscape type are subsequently calculated accordingly:


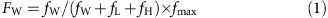


where *F*_W_ is the adjusted regional coverage for wetland thermokarst landscapes that take into account co-occurrence of all thermokarst landscapes, while *f*_W_, *f*_L_ and *f*_H_ are the unadjusted coverages set by the class modes (Table 1) for wetland, lake and hillslope thermokarst landscape coverages respectively, and *f*_max_ is the maximum of *f*_W_, *f*_L_ and *f*_H_. Solving for *F*_L_ and *F*_H_ (adjusted regional lake and hillslope thermokarst landscape coverages) is done analogously. This means, for example, that a region with ‘High' wetland and lake, and ‘Low' hillslope thermokarst landscape coverage will have adjusted coverages of 19, 19 and 2%, respectively, rather than the 40, 40 and 5% if coverages were unadjusted.

### Thermokarst landscape SOC estimates

Estimates of 0–3 m SOC storage for non-thermokarst landscapes and the three thermokarst landscape types within each region are based on available spatial information of 0–3 m SOC storage^1^. This spatial information on SOC storage is scaled using on pedon data stratified both by depth (0–1 m/1–2 m/2–3 m), and by soil type^1^. Partitioning of the total 0–3 m SOC storage within each region of this study uses information of the adjusted thermokarst landscape coverages along with region-specific SOC concentrations (kg C m^−2^) and areal coverages of different soil types (histosols, histels, turbels, orthels and a category of other non-permafrost soils). Wetland thermokarst landscape SOC concentration within a specific region is estimated by using the area-weighted SOC concentrations of histosols and histels within that region. This again acknowledges that wetland thermokarst landscapes are assumed to be a mix of permafrost and non-permafrost peatland ecosystems. Lake and hillslope thermokarst landscape SOC concentrations are both determined by the area-weighted SOC concentrations of histels, turbels and orthels. Non-thermokarst landscape SOC storage is determined by the residual SOC storage after all thermokarst landscape SOC is subtracted from the total regional SOC storage.

### Thermokarst landform database

A database of thermokarst landform study sites (differentiating between thermokarst landforms typical of wetland, lake and hillslope thermokarst landscapes) was compiled from published scientific literature within personal libraries and through Web of Science. Study sites were only included in the database if no other site characteristic of the same thermokarst landscape type was already located within 30 km. This exclusion of nearby sites was done to avoid over-representation of a few intensely studied sites. Hence, the database is not an exhaustive thermokarst literature list, but includes 225 study sites described in 161 studies (Supplementary Tables 4–6). The number and concentrations of study sites within regions of each thermokarst landscape coverage class was calculated (Supplementary Fig. 1).

### Data availability

The maps of thermokarst landscape distribution are published as images and in a polygon data-format through Oak Ridge National Laboratory (ORNL) Distributed Active Archive Center for Biogeochemical Dynamics (DAAC) with the identifier ‘ https://dx.doi.org/10.3334/ORNLDAAC/1332' (ref. 59). Data attributes include polygon total area, thermokarst landscape area of each type, total SOC and SOC stored in each thermokarst landscape type.

## Additional information

**How to cite this article:** Olefeldt, D. *et al*. Circumpolar distribution and carbon storage of thermokarst landscapes. *Nat. Commun.* **7,** 13043 doi: 10.1038/ncomms13043 (2016).

## Supplementary Material

Supplementary Information Supplementary Figures 1-3, Supplementary Tables 1-6 and Supplementary References

## Figures and Tables

**Table 1:** Framework for estimating regional coverage of thermokarst landscapes.

		**Wetland thermokarst landscape**	**Lake thermokarst landscape**	Hillslope thermokarst landscape*
Permafrost zone^25^† (continuous/discontinuous/sporadic/isolated)		−10/−5/0/0	0/0/−10/−20	−15/−15/−25/−35
Ground ice content^25^ (>20%/10–20%/>10%/<10%)		0/0/0/0	0/−10/−10/−30	0/−5/−5/−35
Sedimentary overburden^25^ (thick/thin)		0/−10	0/−40	0/−10
Topography^27^ (flat/undulating/hilly/mountainous)		0/−15/−20/−30	0/−30/−60/−70	−15/0/−5/−15
Histel regional coverage^28^ (>30%/10–30%/<10%)		0/−25/−50	0/0/0	−10/−5/0
Terrestrial ecozone^26^ (boreal/tundra)		0/0	−5/0	−10/0
	*Resulting score*	*Thermokarst landscape coverage:*‡		
	≤25	‘None', 0–1% (0%)		
	30–50	‘Low', 1–10% (5%)		
	55–70	‘Moderate', 10–30% (20%)		
	75–85	‘High', 30–60% (40%)		
	90–100	‘Very High', 60–100% (90%)		

The framework implements a structure where points are subtracted from a maximum score of 100 based on landscape characteristics, as shown in the table. The resulting score yields the coverage classes according to the ranges shown below.

^*^Hillslope thermokarst landscapes are considered to not be able to reach regional ‘Very High' land cover, and thus the maximum score is 85, since at least 15 points are subtracted for the permafrost zone characteristics.

^†^Regions in the southern West Siberian Lowlands are mapped as having only relict permafrost, indicating that permafrost is not affecting the surface soils, and these regions are thus considered to have ‘None' thermokarst landscape coverage.

^‡^Numbers indicate likely ranges and mode (in brackets) for thermokarst landscape coverage within each class.

**Table 2:** Estimates of areas and soil organic carbon storage within the overall study region and in each thermokarst landscape type.

	**Study region**	**Wetland thermokarst landscape**	**Lake thermokarst landscape**	**Hillslope thermokarst landscape**	**Non-thermokarst landscape**
Area (10^6^ km^2^)	18.41	1.43	1.30	0.91	14.77
Area (% of total)	100	7.8	7.1	4.9	80.2
SOC 0–3 m (Pg C)	1,073	164	102	67	740
SOC 0–3 m (% of total)	100	15.3	9.5	6.2	69.0
SOC 0–3 m (kg C m^−2^)	58	114	79	74	50

Average soil organic carbon (SOC) concentration (kg C m^−2^) for each thermokarst landscape is reported.

## References

[b1] Hugelius G. . Estimated stocks of circumpolar permafrost carbon with quantified uncertainty ranges and identified data gaps. Biogeosciences 11, 6573–6593 (2014).

[b2] Grosse G. . Vulnerability of high-latitude soil organic carbon in North America to disturbance. J. Geophys. Res.Biogeosci. 116, G00K06 (2011).

[b3] Schuur E. A. G. . Climate change and the permafrost carbon feedback. Nature 520, 171–179 (2015). 25855454 10.1038/nature14338

[b4] Jorgenson M. T. in *Treatise on Geomorphology* Vol. 8 (eds Giardino, R. & Harbor, J.) 313–324 (Academic Press, 2013).

[b5] Kokelj S. & Jorgenson M. T. Advances in thermokarst research. Permafrost Periglac 24, 108–119 (2013).

[b6] Arneth A. . Terrestrial biogeochemical feedbacks in the climate system. Nat. Geosci. 3, 525–532 (2010).

[b7] Lawrence D. M., Slater A. G. & Swenson S. C. Simulation of present day and future permafrost and seasonally frozen ground conditions in CCSM4. J. Clim. 25, 2207–2225 (2012).

[b8] Harden J. W. . Field information links permafrost carbon to physical vulnerabilities of thawing. Geophys. Res. Lett. 39, L15704 (2012).

[b9] Hayes D. J. . The impacts of recent permafrost thaw on land-atmosphere greenhouse gas exchange. Environ. Res. Lett. 9, 045005 (2014).

[b10] Schaefer K., Lantuit H., Romanovsky V. E., Schuur E. A. G. & Witt R. The impact of the permafrost carbon feedback on global climate. Environ. Res. Lett. 9, 085003 (2014).

[b11] Koven C. D., Lawrence D. M. & Riley W. J. Permafrost carbon-climate feedback is sensitive to deep soil carbon decomposability but not deep soil nitrogen dynamics. Proc. Natl Acad. Sci. USA 112, 3752–3757 (2015). 25775603 10.1073/pnas.1415123112 PMC4378430

[b12] Hopkins D. M. Thaw lakes and thaw sinks in the Imuruk Lake area, Seward Peninsula, Alaska. J. Geol. 57, 119–131 (1949).

[b13] Czudek T. & Demek J. Thermokarst in Siberia and its influence on the development of lowland relief. Quatern. Res. 1, 103–120 (1970).

[b14] Soloviev P. A. Thermokarst phenomena and landforms due to frostheaving in central Yakutia. Biuletyn Peryglacjalny 23, 135–155 (1973).

[b15] Vitt D. H. & Halsey L. A. The bog landforms of continental western Canada in relation to climate and permafrost patterns. Arctic Alpine Res. 26, 1–13 (1994).

[b16] Schuur E. A. G. . The effect of permafrost thaw on old carbon release and net carbon exchange from tundra. Nature 459, 556–559 (2009). 19478781 10.1038/nature08031

[b17] O'Donnell J. A. . The effects of permafrost thaw on soil hydrologic, thermal, and carbon dynamics in an Alaskan peatland. Ecosystems 15, 213–229 (2012).

[b18] Walter Anthony K. M. . A shift of thermokarst lakes from carbon sources to sinks during the Holocene epoch. Nature 511, 452–456 (2014). 25043014 10.1038/nature13560

[b19] Kachurin S. P. Thermokarst on the USSR Terrain Academy of Science Press (1961).

[b20] Jorgenson T. . in *Ninth International Conference on Permafrost, Extended abstracts* (eds Kane, D. L. & Hinkel, K. M.) (Institute of Northern Engineering, University of Alaska, 2008).

[b21] Jones B. M. . Modern thermokarst lake dynamics in the continuous permafrost zone, northern Seward peninsula, Alaska. J. Geophys. Res. Biogeosci 116, G00M03 (2011).

[b22] Balser A. W., Jones J. B. & Gens R. Timing of retrogressive thaw slumps initiation in the Noatak Basin, northwest Alaska, USA. J. Geophys. Res. Earth Surf. 119, F002889 (2014).

[b23] Baltzer J. L., Veness T., Chasmer L. E., Sniderhan A. E. & Quinton W. L. Forest on thawing permafrost: fragmentation, edge effects, and net forest loss. Glob. Chang. Biol. 20, 824–834 (2014). 23939809 10.1111/gcb.12349

[b24] Nelson F. E., Anisimov O. A. & Shiklomanov N. I. Subsidence risk from thawing permafrost. Nature 410, 889–890 (2001). 11309605 10.1038/35073746

[b25] Brown J., Ferrians O., Heginbottom J. A. & Melnikov E. Circum-Arctic Map of Permafrost and Ground-Ice Conditions National Snow and Ice Data Center (2014).

[b26] Olson D. M. . Terrestrial ecoregions of the world: a new map of life on Earth. Bioscience 51, 933–938 (2001).

[b27] Gruber S. Derivation and analysis of a high-resolution estimate of global permafrost zonation. Cryosphere 6, 221–233 (2012).

[b28] Hugelius G. . The Northern Circumpolar Soil Carbon Database: spatially distributed datasets of soil coverage and soil carbon storage in the northern permafrost regions. Earth Syst. Sci. Data 5, 3–13 (2013).

[b29] Permafrost Carbon Network. www.permafrostcarbon.org (2016).

[b30] Kuhry P. Palsa and peat plateau development in the Hudson Bay Lowlands, Canada: timing, pathways and causes. Boreas 37, 316–327 (2008).

[b31] Camill P. Permafrost thaw accelerates in boreal peatlands during late-20th century warming. Clim. Change 1-2, 135–152 (2005).

[b32] Sannel A. B. K. & Kuhry P. Warming-induced destabilization of peat plateau/thermokarst lake complexes. J. Geophys. Res. Biogeosci. 116, G03035 (2011).

[b33] Olefeldt D. . Net carbon accumulation of a high-latitude permafrost palsa mire similar to permafrost-free peatlands. Geophys. Res. Lett. 39, L03501 (2012).

[b34] Grosse G., Jones B. & Arp C. in *Treatise on Geomorphology* Vol. 8 (eds Giardino, R. & Harbor, J.) 325–353 (Academic Press, 2013).

[b35] Osterkamp T. E. . Observations of thermokarst and its impacts on boreal forests in Alaska, U.S.A. Arctic Antarctic Alpine Res. 32, 303–315 (2000).

[b36] Jorgenson M. T. & Osterkamp T. E. Response of boreal ecosystems to varying modes of permafrost degradation. Can. J. For. Res. 35, 2100–2111 (2005).

[b37] Wei Y. . The North American Carbon Program multi-scale synthesis and terrestrial model intercomparison project—Part 2: Environmental driver data. Geosci. Model Dev. 7, 2875–2893 (2014).

[b38] Cohen J. Weighted Kappa: nominal scale agreement with provision for scaled disagreement or partial credit. Psychol. Bull. 70, 213–220 (1969). 10.1037/h0026256 19673146

[b39] Landis J. R. & Koch G. G. The measurement of observer agreement for categorical data. Biometrics 33, 159–174 (1977). 843571

[b40] Naesset E. Use of the weighted Kappa coefficient in classification error assessment of thematic maps. Int. J. Geogr. Inf. Sci. 10, 591–603 (1996).

[b41] Loisel J. . A database and synthesis of northern peatland soil properties and Holocene carbon and nitrogen accumulation. Holocene 24, 1028–1042 (2014).

[b42] Strauss J. . The deep permafrost carbon pool of the Yedoma region in Siberia and Alaska. Geophys. Res. Lett. 40, 6165–6170 (2013). 26074633 10.1002/2013GL058088 PMC4459201

[b43] Niu F., Lin Z., Lu J., Luo J. & Wang H. Assessment of terrain susceptibility to thermokarst lake development along the Qinghai–Tibet engineering corridor, China. Environ. Earth Sci. 73, 5631–5642.

[b44] Coumou D. & Rahmstorf S. A decade of weather extremes. Nat. Clim. Chang. 2, 491–496 (2012).

[b45] Turetsky M. R. . Recent acceleration of biomass burning and carbon losses in Alaskan forests and peatlands. Nat. Geosci. 4, 27–31 (2011).

[b46] Kokelj S. V. . Increased precipitation drives mega slump development and destabilization of ice-rich permafrost terrain, northwestern Canada. Glob. Planet. Chang. 129, 56–68 (2015).

[b47] Collins M. . in *Climate Change 2013: The Physical Science Basis. Contribution of Working Group I to the Fifth Assessment Report of the Intergovernmental Panel on Climate Change* (eds Stocker, T. F. .) (Cambridge University Press, 2013).

[b48] Meehl G. A. . Climate system response to external forcings and climate change projections in CCSM4. J. Clim. 25, 3661–3683 (2012).

[b49] Kunitsky V. V. . Ice-rich permafrost and thermal denudation in the Batagay area (Yana Upland, East Siberia). Kriosfera Zemli. 17, 56–68 (2013).

[b50] Olefeldt D., Turetsky M. R., Crill P. M. & McGuire A. D. Environmental and physical controls on northern terrestrial methane emissions across permafrost zones. Glob. Chang. Biol. 19, 589–603 (2013). 23504795 10.1111/gcb.12071

[b51] Walter K. M., Zimov S., Chanton J. P., Verbyla D. & Chapin F. S. III Methane bubbling from Siberian thaw lakes as a positive feedback to climate warming. Nature 443, 71–75 (2006). 16957728 10.1038/nature05040

[b52] Lamoreaux S. F. & Lafreniere M. J. Seasonal fluxes and age of particulate organic carbon exported from Arctic catchments impacted by localized permafrost slope disturbances. Environ. Res. Lett. 9, 045002 (2014).

[b53] Olefeldt D. & Roulet N. T. Permafrost conditions in peatlands regulate magnitude, timing, and chemical composition of catchment dissolved organic carbon. Glob. Chang. Biol. 20, 3122–3136 (2014). 24753046 10.1111/gcb.12607

[b54] Dutta K., Schuur E. A. G., Neff J. C. & Zimov S. A. Potential carbon release from permafrost soils of Northeastern Siberia. Glob. Chang. Biol. 12, 2336–2351 (2006).

[b55] Vonk J. E. . High biolability of ancient permafrost carbon upon thaw. Geophys. Res. Lett. 40, 2689–2693 (2013).

[b56] Hinkel K. M. . Spatial extent, age, and carbon stocks in drained thaw lake basins on the Barrow Peninsula, Alaska. Arctic Antarctic Alpine Res. 35, 291–300 (2003).

[b57] Treat C. . Effects of permafrost aggradation on peat properties as determined from a pan-arctic synthesis of plant macrofossils. J. Geophys. Res. Biogeosci. 121, 78–94 (2016).

[b58] Schneider von Deimling T. . Observation-based modelling of permafrost carbon fluxes with accounting for deep carbon deposits and thermokarst activity. Biogeosciences 12, 3469–3488 (2014).

[b59] Olefeldt D. . *Arctic Circumpolar Distribution and Soil Carbon of Thermokarst Landscapes, 2015.* https://dx.doi.org/10.3334/ORNLDAAC/1332 (ORNL DAAC, Oak Ridge, TN, USA, 2016). 10.1038/ncomms13043 PMC5062615 27725633

