# Supplementary material for: Circumpolar distribution and carbon storage of thermokarst landscapes
Source: Nat Commun. 2016 Oct 11;7:13043. doi: 10.1038/ncomms13043 (PMC5062615; doi:10.1038/ncomms13043)
Supplement: Supplementary Information — Supplementary Figures 1-3, Supplementary Tables 1-6 and Supplementary References [file ncomms13043-s1.pdf]

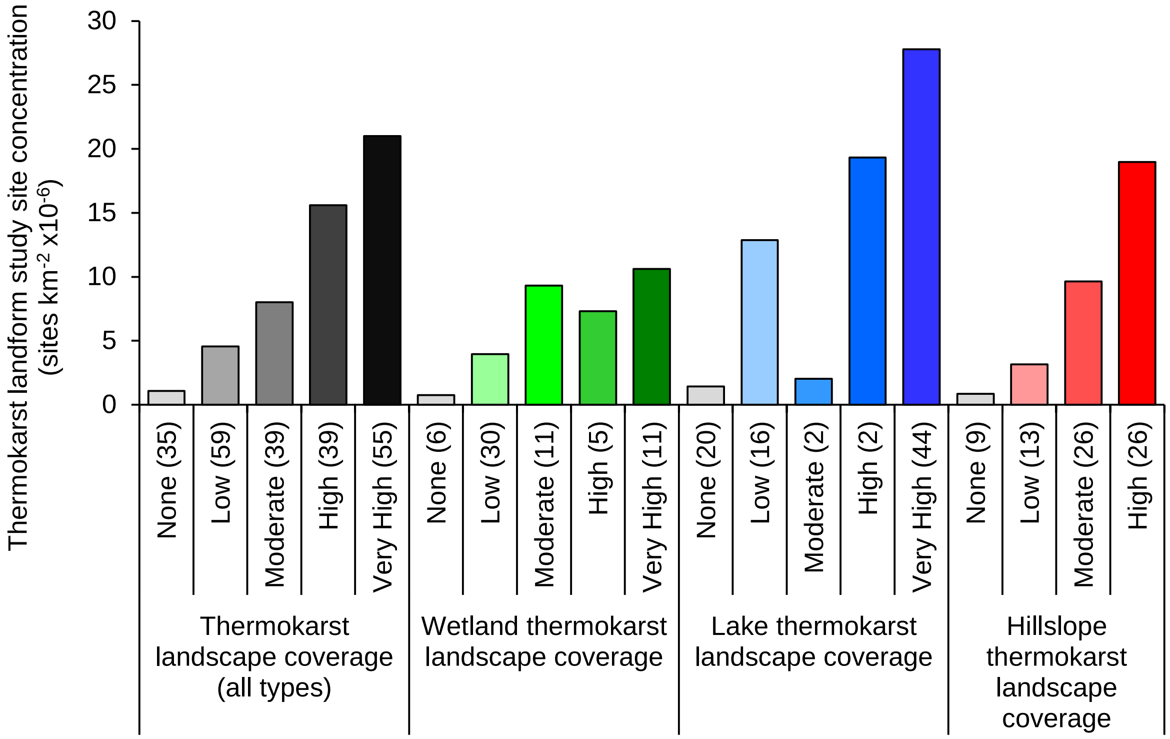

Supplementary Figure 1. Thermokarst study site concentrations in regions mapped with each coverage class. Numbers in brackets indicate the absolute number of study sites within each coverage class. Green, blue, and red shadings indicate site concentrations of wetland, lake and hillslope thermokarst landforms in each thermokarst landscape coverage class, grey shading indicate the overall thermokarst landform study site concentrations in different mapped coverage classes. Coverage is classified as “Very High” (60-100% regional coverage), “High” (30-60%), “Moderate” (10-30%), “Low” (1-10%) and “None” (0-1%).

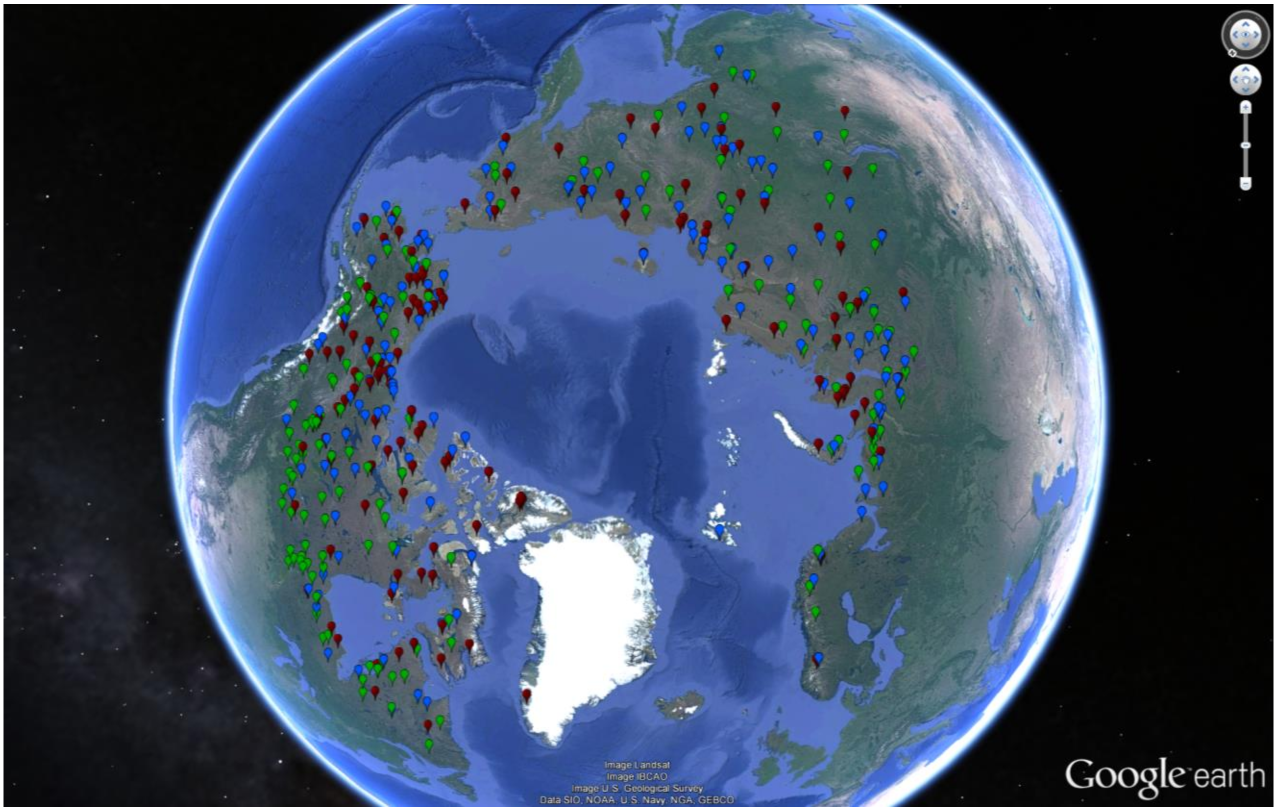

Supplementary Figure 2. Stratified random sites used in the expert assessment of coverage of thermokarst landscapes. Locations of green circles were assessed for wetland thermokarst landscape coverage, blue circles for lake thermokarst landscape coverage, and red circles for hillslope thermokarst landscape coverage. Background map from Google Earth using data sources: Google, Landsat, IBCAO, USGS, NOAA, SIO, US Navy, GEBCO.

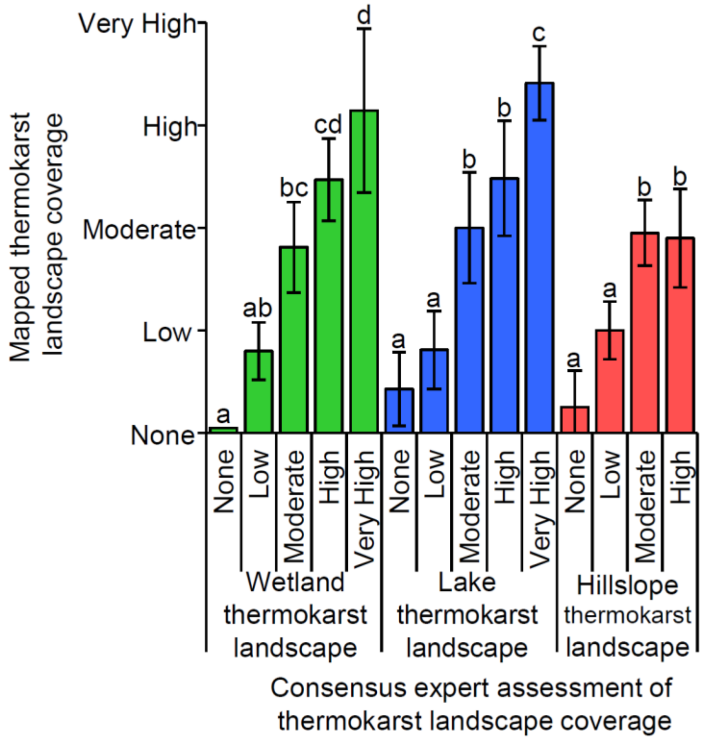

Supplementary Figure 3. Average mapped coverage of thermokarst landscapes (± 2 S.E.) for sites within each coverage class as indicated by the consensus expert assessment. Letters indicate significant differences (p < 0.01) between classes within each thermokarst landscape type, as indicated by an ANOVA followed by Tukey's HSD post-hoc test.

Supplementary Table 1. Confusion matrix for wetland thermokarst landscapes, comparing consensus expert assessment and mapped coverage of thermokarst landscapes. Numbers indicate the percent of total sites (150) in each bin.

		Expert assessment of coverage						
		None	*Low*	*Moderate*	*High*	*Very high*	**User Accuracy**	**Total Accuracy**
Mapped coverage	*None*	11 %	15 %	3 %	2 %	0 %	**36%**	
	Low	0 %	10 %	10 %	6 %	0 %	**38%**	
	Moderate	0 %	4 %	4 %	5 %	2 %	**27%**	
	High	0 %	1 %	3 %	9 %	0 %	**68%**	
	Very high	0 %	1 %	5 %	7 %	3 %	**17%**	
	**Producer Accuracy**	**100%**	**33%**	**16%**	**30%**	**57%**		**37%**

Supplementary Table 2. Confusion matrix for lake thermokarst landscapes, comparing consensus expert assessment and mapped coverage of thermokarst landscapes. Numbers indicate the per cent of total sites (150) in each bin.

		**Expert assessment of coverage**						
		None	*Low*	*Moderate*	*High*	*Very high*	**User Accuracy**	**Total Accuracy**
Mapped coverage	*None*	16 %	14 %	1 %	1 %	1 %	**48%**	
	Low	1 %	4 %	5 %	4 %	1 %	**26%**	
	Moderate	1 %	3 %	3 %	2 %	2 %	**24%**	
	High	1 %	2 %	3 %	4 %	4 %	**30%**	
	Very high	1 %	1 %	2 %	5 %	18 %	**68%**	
	**Producer Accuracy**	**80%**	**17%**	**20%**	**24%**	**69%**		**45%**

Supplementary Table 3. Confusion matrix for hillslope thermokarst landscapes, comparing consensus expert assessment and mapped coverage of thermokarst landscapes. Numbers indicate the per cent of total sites (135) in each bin.

		**Expert assessment of coverage**					
		None	*Low*	*Moderate*	*High*	**User Accuracy**	**Total Accuracy**
Mapped coverage	*None*	7 %	23 %	3 %	1 %	**21%**	
	Low	1 %	7 %	7 %	4 %	**39%**	
	Moderate	1 %	13 %	9 %	4 %	**32%**	
	High	0 %	5 %	11 %	5 %	**24%**	
	**Producer Accuracy**	**83%**	**14%**	**30%**	**37%**		**28%**

Supplementary Table 4. Database of study locations of thermokarst landforms characteristic of wetland thermokarst landscapes. Full reference list follows.

Reference	Latitude	Longitude	Mapped wetland thermokarst landscape coverage
Agafonov et al. 2004[1]	65.05	64.70	Moderate
Allard et al. 1996[2]	55.62	-77.18	Low
Backstrand et al. 2010[3]	68.35	19.03	None
Baltzer et al. 2014[4]	61.30	-121.3	Low
Bauer & Vitt 2011[5]	58.28	-119.37	Low
Beilman & Robinson 2003[6]	64.60	-124.20	High
Beilman & Robinson 2003[6]	62.30	-122.60	Very high
Beilman & Robinson 2003[6]	61.40	-121.81	Low
Beilman & Robinson 2003[6]	57.90	-116.10	Low
Beilman & Robinson 2003[6]	60.50	-120.70	Very high
Beilman & Robinson 2003[6]	55.10	-100.00	Low
Beilman & Robinson 2003[6]	55.80	-107.70	Low
Beilman & Robinson 2003[6]	56.50	-110.00	Low
Beilman & Robinson 2003[6]	57.80	-112.52	Very high
Beilman et al. 2001[7]	56.82	-105.96	Low
Beilman et al. 2001[7]	56.51	-97.00	Low
Blodau et al. 2008[8]	67.50	86.42	Low
Breton et al. 2009[9]	57.50	-76.23	Low
Bubier et al.1995[10]	55.68	-98.42	Moderate
Camill & Clark 1998[11]	54.63	-100.16	Low
Camill & Clark 1998[11]	56.42	-94.33	Moderate
Camill & Clark 1998[11]	55.69	-96.80	Very high
Camill 2005[12]	56.34	-94.71	Very high
Camill 2005[12]	55.73	-97.84	Low
Camill 2005[12]	54.91	-98.63	Low
Camill 2005[12]	54.89	-100.02	Low
Chasmer et al. 2010[13]	61.44	-121.25	Low
Couillard & Payette 1984[14]	58.22	-71.98	Low
Euskirchen et al. 2014[15]	64.70	-148.32	Moderate
Heikkinen et al. 2004[16]	67.38	63.37	High
Hugelius et al. 2011[17]	67.28	62.17	High
Jones et al. 2013[18]	64.68	-148.3	Moderate
Jorgensen 2000[19]	62.85	-143.68	Moderate
Jorgensen et al. 2013[20]	65.19	-156.64	Very high
Jorgensen et al. 2013[20]	63.57	-157.73	Very high
Jorgenson et al. 2001[21]	64.63	-147.70	Moderate
Jorgenson et al. 2001[21]	65.96	-148.41	Low

Supplementary Table 4. Continued.

Reference	Latitude	Longitude	Mapped wetland thermokarst landscape coverage
Kershaw & Gill 1979[22]	63.25	-130.03	None
Laberge & Payette 1995[23]	56.18	-75.92	None
Liblik et al. 1997[24]	61.80	-121.40	Moderate
Luken & Billings 1984[25]	64.87	-147.80	Moderate
Luoto & Seppälä 2003[26]	69.25	26.50	Low
Myers-Smith et al. 2007[27]	64.63	-148.27	High
Nykänen et al. 2003[28]	69.82	27.17	Low
Oberman 2008[29]	67.90	61.95	Moderate
Osterkamp et al. 2000[30]	62.40	-142.70	None
Prater et al. 2007[31]	59.48	-117.20	Very high
Quinton et al. 2009[32]	61.30	-121.30	Low
Sollid & Sorbel 1998[33]	62.10	9.41	None
Thibault & Payette 2009[34]	55.00	-78.00	Low
Thibault & Payette 2009[34]	53.28	-76.37	Low
Thibault & Payette 2009[34]	51.75	-75.00	Low
Thie 1974[35]	54.00	-98.50	Low
Turetsky et al. 2002[36]	55.85	-107.68	Low
Vallee & Payette 2007[37]	57.75	-76.33	Low
Vitt et al. 1994[38]	59.31	-118.94	Very high
Vitt et al. 1994[38]	59.94	-115.80	Very high
Vitt et al. 1994[38]	58.12	-111.81	Low
Vitt et al. 1994[38]	56.14	-98.40	Moderate
Yoshikawa et al. 2003[39]	64.70	-148.10	High
Zuidhoff 2002[40]	66.10	15.50	None

Supplementary Table 5. Database of study locations of thermokarst landforms characteristic of lake thermokarst landscapes. Full reference list follows.

Reference	Latitude	Longitude	Mapped lake thermokarst landscape coverage
Akerman 1992[41]	78.07	13.63	None
Allard et al. 1996[2]	55.62	-77.18	None
Are 1973[42]	62.12	133.45	None
Arp et al. 2011[43]	70.79	-153.07	Very high
Audrey et al. 2011[44]	65.82	75.17	Moderate
Billings & Peterson 1980[45]	71.33	-156.45	Very high
Black & Barksdale 1949[46]	70.50	-156.00	Very high
Blodau et al. 2008[8]	67.50	86.42	Low
Bosikov 1989[47]	61.58	130.67	Very high
Bouchard et al. 2011[48]	55.33	-77.50	None
Breton et al. 2009[9]	73.15	-79.97	None
Breton et al. 2009[9]	57.50	-76.23	None
Breton et al. 2009[9]	56.60	-76.20	None
Brouchkov et al. 2004[49]	62.00	130.20	Low
Burgess et al. 1982[50]	69.48	-134.58	Very high
Burn & Smith 1990[51]	63.58	-135.58	None
Burn 1998[52]	60.85	-135.67	None
Burn 2002[53]	69.47	-134.32	Very high
Dallimore et al. 2000[54]	69.43	-133.97	Very high
Desyatkin et al. 2009[55]	61.67	130.58	Very high
Dredge & Nixon 1979[56]	58.65	-93.80	High
Dyke & Sladen 2010[57]	57.30	-92.90	High
Flessa et al. 2008[58]	67.50	86.43	Low
Grosse et al. 2005[59]	71.78	129.39	Lone
Grosse et al. 2006[60]	73.57	117.30	Very high
Grosse et al. 2008[61]	71.78	129.39	None
Grosse et al. 2008[61]	72.92	123.01	Very high
Grosse et al. 2008[61]	68.76	161.42	Low
Heikkinen et al. 2004[16]	67.38	63.37	Very high
Hill & Solomons 1999[62]	69.72	-134.38	Very high
Hinkel et al. 2012[63]	70.50	-156.83	Very high
Hinkel et al. 2012[63]	70.00	-156.50	Very high
Hopkins 1949[64]	65.58	-163.17	Low
Johnson & Brown 1961[65]	68.30	-133.83	High
Jones et al. 2011[66]	66.50	-164.00	Very high
Jones et al. 2009[67]	70.42	-152.67	Very high
Jorgenson & Osterkamp 2005[68]	64.80	-148.00	None

Supplementary Table 5. Continued.

Reference	Latitude	Longitude	Mapped lake thermokarst landscape coverage
Jorgenson & Shur 2007[69]	70.25	-151.60	Very high
Kallio & Rieger 1969[70]	64.85	-147.85	None
Karlsson et al. 2012[71]	65.00	78.00	Low
Karlsson et al. 2012[71]	64.00	72.00	Very high
Karlsson et al. 2013[72]	65.00	75.00	Low
Katamura et al. 2006[73]	62.15	130.52	Low
Katamura et al. 2006[73]	63.12	130.60	Low
Labrecque et al. 2009[74]	68.00	-139.00	Very high
Lloyd et al. 2003[75]	64.83	-163.70	Low
Luoto & Seppälä 2003[26]	69.25	26.50	None
MacDonald et al. 2012[76]	68.12	-139.72	Very high
MacGraw 2008[77]	70.17	-150.91	Very high
Mackay & Burn 2011[78]	69.43	-124.88	Very high
Mackay 1987[79]	70.01	-129.87	Very high
Mackay 1999[80]	69.84	-131.42	Very high
Mars & Houseknecht 2007[81]	70.85	-153.83	Very high
Marsh 1990[82]	68.32	-133.79	High
Marsh et al. 2009[83]	69.93	-130.60	Very high
Morgenstern et al. 2011[84]	72.72	124.77	Very high
Morgenstern et al. 2011[84]	72.47	128.20	Very high
Morgenstern et al. 2008[85]	73.00	126.00	Very high
Necsoiu et al. 2013[86]	67.10	-158.55	Very high
Osterkamp et al. 2000[30]	64.50	148.90	None
Osterkamp et al. 2000[30]	64.90	147.70	None
Parsekian et al. 2011[87]	66.53	-164.33	Very high
Pelletier 2005[88]	70.50	-154.80	Very high
Pestryakova et al. 2012[89]	63.75	122.70	Very high
Pestryakova et al. 2012[89]	63.35	118.60	High
Plug & West 2009[90]	66.52	-164.48	Very high
Plug & West 2009[90]	68.95	-137.50	Very high
Raynolds et al. 2014[91]	70.28	-148.71	Very high
Riordan et al. 2006[92]	66.84	-149.11	None
Roach et al. 2011[93]	64.38	-158.68	None
Roach et al. 2011[93]	66.07	-149.07	Low
Roach et al. 2011[93]	66.18	-146.07	High
Roach et al. 2011[93]	63.02	-142.12	High
Sannel & Brown 2010[94]	57.88	-94.17	High
Sannel and Kuhry 2011[95]	58.88	-94.17	High
Sannel and Kuhry 2011[95]	67.27	62.13	Very high

Supplementary Table 5. Continued

Reference	Latitude	Longitude	Mapped lake terrain coverage
Sannel and Kuhry 2011[95]	68.47	20.90	Low
Schwamborn et al. 2002[96]	73.33	124.15	Very high
Shilo et al. 2007[97]	68.67	160.98	Very high
Sjöberg et al. 2013[98]	67.05	62.94	Low
Sjöberg et al. 2013[98]	67.16	61.88	Very high
Sjöberg et al. 2013[98]	67.28	62.17	Very high
Smith et al. 2005[99]	65.00	75.00	Low
Sollid & Sorbel 1998[33]	62.10	9.41	None
Stepanenko et al. 2011[100]	68.75	161.40	Low
Taylor et al. 2008[101]	69.25	-134.50	Very high
Tomirdiaro & Ryabchun 1973[102]	65.10	172.25	Moderate
Vallee & Payette 2007[37]	57.75	-76.33	None
van Hardenbroek et al. 2013[103]	70.80	147.60	Very high
Veremeeva & Gubin 2009[104]	69.50	156.00	Very high
Wallace 1948[105]	63.00	-142.00	High

Supplementary Table 6. Database of study locations of thermokarst landforms characteristic of hillslope thermokarst landscapes. Full reference list follows.

Reference	Latitude	Longitude	Mapped hillslope thermokarst landscape coverage
Astakhov & Isayeva 1988[106]	66.59	86.57	Moderate
Balser et al. 2009[107]	68.24	-158.16	Low
Balser et al. 2009[107]	68.02	-159.21	High
Balser et al. 2009[107]	68.04	-157.82	Low
Balser et al. 2014[108]	67.92	-156.85	Low
Belshe et al. 2013[109]	63.87	-149.25	Low
Biskaborn et al. 2013[110]	71.30	125.53	High
Burn & Zhang 2009[111]	69.57	-138.88	High
Burn 2000[112]	63.58	-135.58	None
Carter & Galloway 1981[113]	69.50	-151.95	High
Couture & Riopel 2008[114]	68.35	-133.72	Low
Dallimore et al. 2000[54]	69.43	-133.97	Moderate
Dallimore et al. 1996[115]	69.62	-131.26	Moderate
de Krom 1990[116]	69.59	-139.07	High
Deison et al. 2012[117]	68.50	-133.72	Moderate
Dredge et al. 1999[118]	67.63	-111.90	Low
Fedorov & Konstantinov 1998[119]	61.76	130.47	Moderate
Fortier et al. 2007[120]	73.17	-80.08	Moderate
French 1974[121]	72.68	-119.26	High
French 1975[122]	71.99	-125.23	High
Grosse et al. 2005[59]	71.78	129.39	None
Günther et al. 2012[123]	71.88	132.58	High
Günther et al. 2012[123]	71.42	132.10	High
Günther et al. 2012[123]	71.59	132.22	High
Hegginbottom 1978[124]	75.93	-107.90	High
Hyatt 1992[125]	66.15	-65.68	None
Jolivel & Allard 2013[126]	56.62	-76.53	Moderate
Kokelj et al. 2005[127]	68.97	-133.82	Moderate
Kokelj et al. 2005[127]	69.27	-134.58	Moderate
Kokelj et al. 2013[128]	67.32	-135.26	Moderate
Lacelle et al. 2010[129]	68.15	-135.58	None
Lantuit & Pollard 2008[130]	69.57	-139.08	High
Lantuit et al. 2012[131]	69.57	-138.92	High
Lantuit et al. 2012[131]	69.08	-137.90	Moderate
Lantz & Kokelj 2008[132]	69.03	-134.16	Moderate
Lantz et al. 2009[133]	69.55	-135.00	Moderate
Lantz et al. 2009[133]	68.27	-133.00	Moderate

Supplementary Table 6. Continued.

Reference	Latitude	Longitude	Mapped hillslope thermokarst landscape coverage
Lawson 1986[134]	69.79	-155.54	Moderate
Leibman 1995[135]	70.41	68.52	Moderate
Leibman et al. 2008[136]	69.75	62.00	Moderate
Lewellen 1970[137]	71.25	-156.70	Moderate
Lewkowicz 1987[138]	71.70	-124.03	High
Lewkowicz 1990[139]	79.97	-85.67	Low
Lewkowicz 1990[139]	79.97	-84.47	High
Lewkowicz 1990[139]	79.70	-84.38	High
Lewkowicz & Harris 2005a[140]	64.28	-124.67	Low
Lewkowicz & Harris 2005b[141]	65.03	-126.13	Moderate
Lewkowicz & Harris 2005b[141]	64.28	-124.47	Low
Lewkowicz & Harris 2005b[141]	79.98	-85.65	Low
Lewkowicz & Harris 2005b[141]	79.97	-84.30	High
Lewkowicz 2007[142]	79.70	-84.42	High
Liljedahl et al. 2007[143]	65.44	-164.58	High
Lipovsky & Huscroft 2006[144]	62.20	-133.37	None
Lyle et al. 2005[145]	62.17	-134.70	None
MacGraw 2008[77]	70.17	-150.91	High
Mackay 1970[146]	62.17	-134.70	None
Malone et al. 2013[147]	67.25	-135.22	Moderate
Mars & Houseknecht 2007[81]	70.85	-153.82	Moderate
McRoberts & Morgenstern 1974[148]	65.95	-128.67	None
McRoberts & Morgenstern 1974[148]	62.09	-122.01	None
Rudy et al. 2013[149]	74.90	-109.58	Low
Seppälä 1997[150]	62.10	-74.52	Low
Sidorchuk & Matveev 1994[151]	70.58	67.93	Moderate
Singhroy et al. 2010[152]	67.52	-130.79	Moderate
Smirnov 1986[153]	71.16	67.02	Moderate
Streletskyi et al. 2008[154]	69.00	-149.00	High
Toniolo et al. 2009[155]	65.17	-147.50	Moderate
Vogel et al. 2009[156]	63.88	-149.25	Low
Wang et al. 2009[157]	68.20	-132.75	Moderate

Supplementary References (Study site database):

[1]Agafonov, L., Strunk, H. & Nuber, T. Thermokarst dynamics in Western Siberia: Insights from dendrochronological research. *Palaeogeogr. Palaeoclimatol. Palaeoecol.* **209,** 183–196 (2004).

[2]Allard, M., Caron, S. & Begin, Y. Climatic and ecological controls on ice segregation and thermokarst: The case history of a permafrost plateau in northern Quebec. *Permafr. Periglac. Process.* **7,** 207–227 (1996).

[3]Bäckstrand, K. *et al.* Annual carbon gas budget for a subarctic peatland, northern Sweden. Biogeosciences **7,** 95–108 (2010).

[4]Baltzer, J. L., Veness, T., Chasmer, L. E., Sniderhan, A. E. & Quinton, W. L. Forests on thawing permafrost: Fragmentation, edge effects, and net forest loss. *Glob. Chang. Biol.* **20,** 824–834 (2014).

[5]Bauer, I. E. & Vitt, D. H. Peatland dynamics in a complex landscape: Development of a fen-bog complex in the Sporadic Discontinuous Permafrost zone of northern Alberta, Canada. *Boreas* **40,** 714–726 (2011).

[6]Beilman, D. W. & Robinson, S. D. Peatland permafrost thaw and landform type along a climatic gradient. *Proceedings of the 8th International Conference on Permafrost* 61–65 (2003).

[7]Beilman, D. W., Vitt, D. H. & Halsey, L. A. Permafrost in western localized peatlands Canada: definition, distributions, and degradation. *Arctic, Antarct. Alp. Res.* **33,** 70–77 (2001).

[8]Blodau, C. *et al.* A snapshot of CO2 and CH4 evolution in a thermokarst pond near Igarka, northern Siberia. *J. Geophys. Res.* **113,** 1–8 (2008).

[9]Breton, J., Vallières, C. & Laurion, I. Limnological properties of permafrost thaw ponds in northeastern Canada. *Can. J. Fish. Aquat. Sci.* **66,** 1635–1648 (2009).

[10]Bubier, J. L., Moore, T. R., Bellisario, L., Comer, N. T. & Crill, P. M. Ecological controls on methane emissions from a northern peatland complex in the zone of discontinuous permafrost, Manitoba, Canada. *Global Biogeochem. Cycles* **9,** 455–470 (1995).

[11]Camill, P. & Clark, J. S. Climate change disequilibrium of boreal permafrost peatlands caused by local processes. *Am. Nat.* **151,** 207–222 (1998).

[12]Camill, P. Permafrost thaw accelerates in boreal peatlands during late-20th century climate warming. *Clim. Change* **68,** 135–152 (2005).

[13]Chasmer, L., Hopkinson, C. & Quinton, W. Quantifying errors in discontinuous permafrost plateau change from optical data, Northwest Territories, Canada: 1947-2008. *Can. J. Remote Sens.* **36,** S211–S223 (2010).

[14]Couillard, L. & Payette, S. Evolution Holocene d'une tourbiere a pergelisol (Quebec nordique). *Can. J. Bot.* **63,** 1104–1121 (1985).

[15]Euskirchen, E. S., Edgar, C. W., Turetsky, M. R., Waldrop, M. P. & Harden, J. W. Differential response of carbon fluxes to climate in three peatland ecosystems that vary in the presence and stability of permafrost. *J. Geophys. Res. Biogeosciences* **119,** 1576–1595 (2014).

[16]Heikkinen, J. E. P., Virtanen, T., Huttunen, J. T., Elsakov, V. & Martikainen, P. J. Carbon balance in east European tundra. *Global Biogeochem. Cycles* **18,** GB1023 (2004).

[17]Hugelius, G. *et al.* High-resolution mapping of ecosystem carbon storage and potential effects of permafrost thaw in periglacial terrain, European Russian Arctic. *J. Geophys. Res. Biogeosciences* **116,** G03024 (2011).

[18]Jones, M. C., Booth, R. K., Yu, Z. & Ferry, P. A 2200-year record of permafrost dynamics and carbon cycling in a collapse-scar bog, interior Alaska. *Ecosystems* **16,** 1–19 (2013).

[19]Jorgenson, M.T. Hierarchical organization of ecosystems at multiple scales on the Yukon-Kuskokwim delta, Alaska, U.S.A. *Arctic, Antarct. Alp. Res.* **32,** 221-239 (2000).

[20]Jorgenson, M. *et al.* Reorganization of vegetation, hydrology and soil carbon after permafrost degradation across heterogeneous boreal landscapes. *Environ. Res. Lett.* **8,** 035017 (2013).

[21]Jorgenson, M. T., Racine, C. H., Walters, J. C. & Osterkamp, T. E. Permafrost degradation and ecological changes associated with a warming climate in central Alaska. *Clim. Change* **48,** 551–579 (2001).

[22]Kershaw, G. P. & Gill, D. Growth and decay of palsas and peat plateaus in the Macmillan Pass - Tsichu River area, Northwest Territories, Canada. *Can. J. Earth Sci.* **16,** 1362–1374 (1979).

[23]Laberge, M. & Payette, S. Long-term monitoring of permafrost change in a palsa peatland in northern Quebec, Canada: 1983-1993. *Arct. Alp. Res.* **27,** 167–171 (1995).

[24]Liblik, L. K., Moore, T. R., Bubier, J. L. & Robinson, S. D. Methane emissions from wetlands in the zone of discontinuous permafrost: Fort Simpson, Northwest Territories, Canada. *Global Biogeochem. Cycles* **11,** 485–494 (1997).

[25]Luken, J. O. & Billings, W. D. Changes in bryophyte production associated with a thermokarst erosion cycle in a subarctic bog. *Lindbergia* **9,** 163–168 (1984).

[26]Luoto, M. & Seppälä, M. Thermokarst ponds as indicators of the former distribution of palsas in finnish lapland. *Permafr. Periglac. Process.* **14,** 19–27 (2003).

[27]Myers-Smith, I. H., McGuire, A. D., Harden, J. W. & Chapin, F. S. Influence of disturbance on carbon exchange in a permafrost collapse and adjacent burned forest. *J. Geophys. Res. Biogeosciences* **112,** G04017 (2007).

[28]Nykänen, H., Heikkinen, J. E. P., Pirinen, L., Tiilikainen, K. & Martikainen, P. J. Fluxes on a subarctic palsa mire during climatically different years. *Global Biogeochem. Cycles* **17,** 1018 (2003).

[29]Oberman, N. Contemporary permafrost degradation of northern European Russia. in *Proc. 9th Int. Conf. Permafr.* (Kane, D. & Hinkel, K. M.) 1305–1310 (2008).

[30]Osterkamp, T. E. *et al.* Observations of thermokarst and its impact on boreal forests in Alaska, USA. *Arctic, Antarct. Alp. Res.* **32,** 303–315 (2000).

[31]Prater, J. L., Chanton, J. P. & Whiting, G. J. Variation in methane production pathways associated with permafrost decomposition in collapse scar bogs of Alberta, Canada. *Global Biogeochem. Cycles* **21,** GB4004 (2007).

[32]Quinton, W. L., Hayashi, M. & Chasmer, L. E. Peatland Hydrology of Discontinuous Permafrost in the Northwest Territories: Overview and Synthesis. *Can. Water Resour. J.* **34,** 311–328 (2009).

[33]Sollid, J. L. & Sorbel, L. Palsa bogs as a climate indicator: Examples from Dovrefjell, southern Norway. *Ambio* **27,** 287–291 (1998).

[34]Thibault, S. & Payette, S. Recent permafrost degradation in bogs of the James Bay area, northern Quebec, Canada. *Permafr. Periglac. Process.* **20,** 383–389 (2009).

[35]Thie, J. Distribution and thawing of permafrost in the sourthern part of the discontinuous permafrost zone in Manitoba. *Arctic* **27,** 189–200 (1974).

[36]Turetsky, M. R., Wieder, R. K. & Vitt, D. H. Boreal peatland C fluxes under varying permafrost regimes. *Soil Biol. Biochem.* **34,** 907–912 (2002).

[37]Vallée, S. & Payette, S. Collapse of permafrost mounds along a subarctic river over the last 100 years (northern Québec). *Geomorphology* **90,** 162–170 (2007).

[38]Vitt, D. H., Halsey, L. A. & Zoltai, S. C. The bog landforms of continental Western Canada in relation to climate and permafrost patterns. *Arct. Alp. Res.* **26,** 1–13 (1994).

[39]Yoshikawa, K., Bolton, W. R., Romanovsky, V. E., Fukuda, M. & Hinzman, L. D. Impacts of wildfire on the permafrost in the boreal forests of Interior Alaska. *J. Geophys. Res.* **108,** 8148 (2003).

[40]Zuidhoff, F. S. Recent decay of a single palsa in relation to weather conditions between 1996 and 2000 in Laivadalen, northern Sweden. *Geogr. Ann. Ser. A, Phys. Geogr.* **84A,** 103–111 (2002).

[41]Åkerman, H. J. Hydrographic characteristics of the Strokdammane Plain, west Spitsbergen, Svalbard. *Geogr. Ann. Ser. A, Phys. Geogr.* **74,** 169–182 (1992).

[42]Are, F. E. Development of thermokarst lakes in Central Yakutia. in *Proc. 2nd Int. Permafr. Conf.* (1973).

[43]Arp, C. D., Jones, B. M., Urban, F. E. & Grosse, G. Hydrogeomorphic processes of thermokarst lakes with grounded-ice and floating-ice regimes on the Arctic coastal plain, Alaska. *Hydrol. Process.* **25,** 2422–2438 (2011).

[44]Audry, S., Pokrovsky, O. S., Shirokova, L. S., Kirpotin, S. N. & Dupré, B. Organic matter mineralization and trace element post-depositional redistribution in Western Siberia thermokarst lake sediments. *Biogeosciences* **8,** 3341–3358 (2011).

[45]Billings, W. D. & Peterson, K. M. Vegetational change and ice-wedge polygons through the thaw-lake cycle in Arctic Alaska. *Arct. Alp. Res.* **12,** 413–432 (1980).

[46]Black, R. F. & Barksdale, W. L. Oriented lakes of northern Alaska. *J. Geol.* **57,** 105–118 (1949).

[47]Bosikov, N. P. The intensity of destruction of fields in inter-alas landscapes, central Yakutia. *Polar Geogr. Geol.* **13,** 149–154 (1989).

[48]Bouchard, F., Francus, P., Pienitz, R. & Laurion, I. Sedimentology and geochemistry of thermokarst ponds in discontinuous permafrost, subarctic Quebec, Canada. *J. Geophys. Res. Biogeosciences* **116,** 1–14 (2011).

[49]Brouchkov, A., Fukuda, M., Fedorov, A., Konstantinov, P. & Iwahana, G. Thermokarst as a short-term permafrost disturbance, Central Yakutia. *Permafr. Periglac. Process.* **15,** 81–87 (2004).

[50]Burgess, M., Judge, A., Taylor, A. & Allen, V. Ground temperature studies of permafrost growth at a drained lake site, Mackenzie Delta. in *Proc. 4th Can. Permafr. Conf.* 3–11 (1982).

[51]Burn, C. R. & Smith, M. W. Development of thermokarst lakes during the Holocene at sites near Mayo, Yukon territory. *Permafr. Periglac. Process.* **1,** 161–176 (1990).

[52]Burn, C. R. The response (1958-1997) of permafrost and near-surface ground temperatures to forest fire, Takhini River valley, southern Yukon Territory. *Can. J. Earth Sci.* **35,** 184–199 (1998).

[53]Burn, C. R. Tundra lakes and permafrost, Richards Island, western Arctic coast, Canada. *Can. J. Earth Sci.* **39,** 1281–1298 (2002).

[54]Dallimore, A., Schröder-Adams, C. J. & Dallimore, S. R. Holocene environmental history of thermokarst lakes on Richards Island, Northwest Territories, Canada: thecamoebians as paleolimnological indicators. *J. Paleolimnol.* **23,** 261–283 (2000).

[55]Desyatkin, A. R. *et al.* CH4 emission from different stages of thermokarst formation in Central Yakutia, East Siberia. *Soil Sci. Plant Nutr.* **55,** 558–570 (2009).

[56]Dredge, L. A. & Nixon, F. M. in *Curr. Res. Part C* 23–26 (Geological Survey of Canada, 1979).

[57]Dyke, L. D. & Sladen, W. E. Permafrost and peatland evolution in the northern Hudson Bay lowland, Manitoba. *Arctic* **63,** 429–441 (2010).

[58]Flessa, H. *et al.* Landscape controls of CH4 fluxes in a catchment of the forest tundra ecotone in northern Siberia. *Glob. Chang. Biol.* **14,** 2040–2056 (2008).

[59]Grosse, G., Schirrmeister, L., Kunitsky, V. V. & Hubberten, H. W. The use of CORONA images in remote sensing of periglacial geomorphology: An illustration from the NE Siberian coast. *Permafr. Periglac. Process.* **16,** 163–172 (2005).

[60]Grosse, G., Schirrmeister, L. & Malthus, T. J. Application of Landsat-7 satellite data and a DEM for the quantification of thermokarst-affected terrain types in the periglacial Lena-Anabar coastal lowland. *Polar Res.* **25,** 51–67 (2006).

[61]Grosse, G. *et al.* Distribution of thermokarst lakes and ponds at three yedoma sites in Siberia. in *Proc. 9th Int. Conf. Permafr.* (Kane, D. & Hinkel, K. M.) 551–556 (2008).

[62]Hill, P. R. & Solomons, S. Geomorphologic and sedimentary evolution of a transgressive thermokarst coast, Mackenzie Delta Region, Canadian Beaufort Sea. *J. Coast. Res.* **15,** 1011–1029 (1999).

[63]Hinkel, K. M. *et al.* Thermokarst lakes on the arctic coastal plain of Alaska: geomorphic controls on bathymetry. *Permafr. Periglac. Process.* **23,** 218–230 (2012).

[64]Hopkins, D. M. Thaw lakes and thaw sinks in the Imuruk Lake area, Seward Peninsula, Alaska. *J. Geol.* **57,** 119–131 (1949).

[65]Johnson, G. H. & Brown, R. J. E. Effect of a lake on distribution of permafrost in the Mackenzie River Delta. *Nature* **192,** 251–252 (1961).

[66]Jones, B. M. *et al.* Modern thermokarst lake dynamics in the continuous permafrost zone, northern Seward Peninsula, Alaska. *J. Geophys. Res. Biogeosciences* **116,** G00M03 (2011).

[67]Jones, B. M. *et al.* Arctic lake physical processes and regimes with implications for winter water availability and management in the National Petroleum Reserve Alaska. *Environ. Manage.* **43,** 1071–1084 (2009).

[68]Jorgenson, M. T. & Osterkamp, T. E. Response of boreal ecosystems to varying modes of permafrost degradation. *Can. J. For. Res.* **35,** 2100–2111 (2005).

[69]Jorgenson, M. T. & Shur, Y. Evolution of lakes and basins in northern Alaska and discussion of the thaw lake cycle. *J. Geophys. Res. Earth Surf.* **112,** F02S17 (2007).

[70]Kallio, A. & Rieger, S. Recession of permafrost in a cultivated soil of interior Alaska. *Soil Sci. Soc. Am. J.* **33,** 430–432 (1969).

[71]Karlsson, J. M., Lyon, S. W. & Destouni, G. Thermokarst lake, hydrological flow and water balance indicators of permafrost change in Western Siberia. *J. Hydrol.* **464-465,** 459–466 (2012).

[72]Karlsson, J. M., Lyon, S. W. & Destouni, G. Temporal behavior of lake size-distribution in a thawing permafrost landscape in northwestern Siberia. *Remote Sens.* **6,** 621–636 (2013).

[73]Katamura, F., Fukuda, M., Bosikov, N. P. & Desyatkin, R. V. Charcoal records from thermokarst deposits in central Yakutia, eastern Siberia: Implications for forest fire history and thermokarst development. *Quat. Res.* **71,** 36–40 (2009).

[74]Labrecque, S., Lacelle, D., Duguay, C. R., Lauriol, B. & Hawkings, J. Contemporary (1951-2001) evolution of lakes in the Old Crow Basin, northern Yukon, Canada: Remote sensing, numerical modeling, and stable isotope analysis. *Arctic* **62,** 225–238 (2009).

[75]Lloyd, A. H., Yoshikawa, K., Fastie, C. L., Hinzman, L. & Fraver, M. Effects of permafrost degradation on woody vegetation at arctic treeline on the Seward Peninsula, Alaska. *Permafr. Periglac. Process.* **14,** 93–101 (2003).

[76]Macdonald, L. a. *et al.* Tracking hydrological responses of a thermokarst lake in the Old Crow Flats (Yukon Territory, Canada) to recent climate variability using aerial photographs and paleolimnological methods. *Hydrol. Process.* **26,** 117–129 (2012).

[77]MacGraw, M. The degradation of ice wedges in the Colville River Delta and their role in pond drainage. in *Proc. 9th Int. Conf. Permafr.* (Kane, D. L. & Hinkel, K. M.) 1161–1166 (2008).

[78]Mackay, J. R. & Burn, C. R. A Century (1910-2008) of change in a collapsing pingo, Parry Peninsula, western Arctic coast, Canada. *Permafr. Periglac. Process.* **22,** 266–272 (2011).

[79]Mackay, J. R. Some mechanical aspects of pingo growth and failure, western Arctic coast, Canada. *Can. J. Earth Sci.* **24,** 1108–1119 (1987).

[80]Mackay, J. R. Periglacial features developed on the exposed lake bottoms of seven lakes that drained rapidly after 1950, Tuktoyaktuk Peninsula area, western Arctic coast, Canada. *Permafr. Periglac. Process.* **10,** 39–63 (1999).

[81]Mars, J. C. & Houseknecht, D. W. Quantitative remote sensing study indicates doubling of coastal erosion rate in past 50 yr along a segment of the Arctic coast of Alaska. *Geology* **35,** 583–586 (2007).

[82]Marsh, P. Permafrost and lakes in the Mackenzie Delta. in *Proc. 4th Can. Permafr. Conf.* 131–136 (1990).

[83]Marsh, P., Russell, M., Pohl, S., Haywood, H. & Onclin, C. Changes in thaw lake drainage in the Western Canadian Arctic from 1950 to 2000. *Hydrol. Process.* **23,** 145–158 (2009).

[84]Morgenstern, A., Grosse, G., Günther, F., Fedorova, I. & Schirrmeister, L. Spatial analyses of thermokarst lakes and basins in Yedoma landscapes of the Lena Delta. *Cryosphere* **5,** 849–867 (2011).

[85]Morgenstern, A., Grosse, G. & Schirrmeister, L. Genetic, morphological, and statistical characterization of lakes in the permafrost-dominated Lena Delta. in *Proc. 9th Int. Conf. Permafr.* (Kane, D. & Hinkel, K. M.) 1239–1244 (2008).

[86]Necsoiu, M., Dinwiddie, C. L., Walter, G. R., Larsen, A. & Stothoff, S. A. Multi-temporal image analysis of historical aerial photographs and recent satellite imagery reveals evolution of water body surface area and polygonal terrain morphology in Kobuk Valley National Park, Alaska. *Environ. Res. Lett.* **8,** 025007 (2013).

[87]Parsekian, A. D. *et al.* Expansion rate and geometry of floating vegetation mats on the margins of thermokarst lakes, northern Seward Peninsula, Alaska, USA. *Earth Surf. Process. Landforms* **36,** 1889–1897 (2011).

[88]Pelletier, J. D. Formation of oriented thaw lakes by thaw slumping. *J. Geophys. Res. Earth Surf.* **110,** F02018 (2005).

[89]Pestryakova, L. A., Herzschuh, U., Wetterich, S. & Ulrich, M. Present-day variability and Holocene dynamics of permafrost-affected lakes in central Yakutia (eastern Siberia) inferred from diatom records. *Quat. Sci. Rev.* **51,** 56–70 (2012).

[90]Plug, L. J. & West, J. J. Thaw lake expansion in a two-dimensional coupled model of heat transfer, thaw subsidence, and mass movement. *J. Geophys. Res. Earth Surf.* **114,** F01002 (2009).

[91]Raynolds, M. K. *et al.* Cumulative geoecological effects of 62 years of infrastructure and climate change in ice-rich permafrost landscapes, Prudhoe Bay Oilfield, Alaska. *Glob. Chang. Biol.* **20,** 1211–1224 (2014).

[92]Riordan, B., Verbyla, D. & McGuire, A. D. Shrinking ponds in subarctic Alaska based on 1950-2002 remotely sensed images. *J. Geophys. Res. Biogeosciences* **111,** G04002 (2006).

[93]Roach, J., Griffith, B., Verbyla, D. & Jones, J. Mechanisms influencing changes in lake area in Alaskan boreal forest. *Glob. Chang. Biol.* **17,** 2567–2583 (2011).

[94]Sannel, A. B. K. & Brown, I. A. High-resolution remote sensing identification of thermokarst lake dynamics in a subarctic peat plateau complex. *Can. J. Remote Sens.* **36,** S26–S40 (2010).

[95]Sannel, A. B. K. & Kuhry, P. Warming-induced destabilization of peat plateau/thermokarst lake complexes. *J. Geophys. Res. Biogeosciences* **116,** G03035 (2011).

[96]Schwamborn, G. J., Dix, J. K., Bull, J. M. & Rachold, V. High-resolution seismic and ground penetrating radar-geophysical profiling of a thermokarst lake in the western Lena Delta, northern Siberia. *Permafr. Periglac. Process.* **13,** 259–269 (2002).

[97]Shilo, N. A., Lozhkin, A. V. & Anderson, P. M. Radiocarbon dates of evolution cycles of thermokarst lakes on the Kolyma Lowland. *Dokl. Earth Sci.* **413,** 259–261 (2007).

[98]Sjöberg, Y., Hugelius, G. & Kuhry, P. Thermokarst lake morphometry and erosion features in two peat plateau areas of northeast European Russia. *Permafr. Periglac. Process.* **24,** 75–81 (2013).

[99]Smith, L. C., Sheng, Y., MacDonald, G. M. & Hinzman, L. D. Disappearing Arctic lakes. *Science.* **308,** 1429 (2005).

[100]Stepanenko, V. M., Machul'skaya, E. E., Glagolev, M. V. & Lykossov, V. N. Numerical modeling of methane emissions from lakes in the permafrost zone. *Izv. Atmos. Ocean. Phys.* **47,** 252–264 (2011).

[101]Taylor, A. E., Dallimore, S. R. & Wright, J. F. Thermal impact of Holocene lakes on a permafrost landscape, Mackenzie Delta, Canada. in *Proc. 9th Int. Conf. Permafr.* (Kane, D. & Hinkel, K. M.) 1757–1762 (2008).

[102]Tomirdiaro, S. V & Ryabchun, V. K. TomirdiaroLake thermokarst on the Lower Anadyr Lowland. in *Proc. 2th Int. Permafr. Conf.* 94–100 (National Academy of Sciences, Washington, D. C., 1973).

[103]van Hardenbroek, M. *et al.* Evidence for past variations in methane availability in a Siberian thermokarst lake based on δ13C of chitinous invertebrate remains. *Quat. Sci. Rev.* **66,** 74–84 (2013).

[104]Veremeeva, A. & Gubin, S. Modern Tundra Landscapes of the Kolyma Lowland and their Evolution in the Holocene. *Permafr. Periglac. Process.* **20,** 399–406 (2009).

[105]Wallace, R. E. Cave-in lakes in the Nabesna, Chisana, and Tanana River Valleys, eastern Alaska. *J. Geol.* **56,** 171–181 (1948).

[106]Astakhov, V. I. & Isayeva, L. L. The 'ice hill': An example of 'retarded deglaciation' in Siberia. *Quat. Sci. Rev.* **7,** 29–40 (1988).

[107]Balser, A. W., Gooseff, M. N., Jones, J. B. & Bowden, W. B. *Thermokarst distribution and relationships to landscape characteristics in the Feniak Lake region, Noatak National Preserve, Alaska*. (2009).

[108]Balser, A. W., Jones, J. B. & Gens, R. Timing of retrogressive thaw slump initiationin the Noatak Basin, northwest Alaska, USA. *J. Geophys. Res. Earth Surf.* **119,** 1106–1120 (2014).

[109]Belshe, E. F., Schuur, E. A. G. & Grosse, G. Quantification of upland thermokarst features with high resolution remote sensing. *Environ. Res. Lett.* **8,** 035016 (2013).

[110]Biskaborn, B. K., Herzschuh, U., Bolshiyanov, D. Y., Schwamborn, G. & Diekmann, B. Thermokarst processes and depositional events in a Tundra Lake, northeastern Siberia. *Permafr. Periglac. Process.* **24,** 160–174 (2013).

[111]Burn, C. R. & Zhang, Y. Permafrost and climate change at Herschel Island (Qikiqtaruq), Yukon Territory, Canada. *J. Geophys. Res. Earth Surf.* **114,** F02001 (2009).

[112]Burn, C. R. The thermal regime of a retrogressive thaw slump near Mayo, Yukon Territory. *Can. J. Earth Sci.* **37,** 967–981 (2000).

[113]Carter, L. D. & Galloway, J. P. Earth flows along henry creek, northern Alaska. *Arctic* **34,** 325–328 (1981).

[114]Couture, R. & Riopel, S. Regional landslide susceptibility mapping and inventorying in the Mackenzie Valley, Northwest Territories. in *Proc. 4th Can. Conf. Geohazards* (2008).

[115]Dallimore, S. R., Wolfe, S. A. & Solomon, S. M. Influence of ground ice and permafrost on coastal evolution, Richards Island, Beaufort Sea coast, N.W.T. *Can. J. Earth Sci.* **33,** 664–675 (1996).

[116]de Krom, V. A geomorphic investigation of retrogressive thaw slumps and active layer slides on Herschel Island, Yukon Territory. (1990).

[117]Deison, R. *et al.* Spatial and temporal assessment of mercury and organic matter in thermokarst affected lakes of the Mackenzie Delta uplands, NT, Canada. *Environ. Sci. Technol.* **46,** 8748–8755 (2012).

[118]Dredge, L. A., Kerr, D. E. & Wolfe, S. A. Surficial materials and related ground ice conditions, Slave Province, N.W.T., Canada. *Can. J. Earth Sci.* **36,** 1227–1238 (1999).

[119]Federov, A. & Konstantinov, P. Observations of surface dynamics with thermokarst initiation, Yukechi site, Central Yakutia. in *Proc. 7th Int. Permafr. Conf.* 139–243 (1998).

[120]Fortier, D., Allard, M. & Shur, Y. Observation of rapid drainage system development by thermal erosion of ice wedges on Bylot Island, Canadian Arctic Archipelago. *Permafr. Periglac. Process.* **18,** 229–243 (2007).

[121]French, H. M. Active thermokarst processes, eastern Banks Island, western Canadian Arctic. *Can. J. Earth Sci.* **11,** 785–794 (1974).

[122]French, H. M. Man-Induced Thermokarst, Sachs Harbour Airstrip, Banks Island, Northwest Territories. *Can. J. Earth Sci.* **12,** 132–144 (1975).

[123]Günther, F., Overduin, P. P., Sandakov, A. V, Grosse, G. & Grigoriev, M. N. Thermo-erosion along the Yedoma coast of the Buor Khaya Peninsula, Laptev Sea, east Siberia. in *Proc. 10th Int. Conf. Permafr.* **2,** 137–142 (2012).

[124]Heginbottom, J. A. in *Curr. Res. Part A* 525–526 (Geological Survey of Canada, 1978).

[125]Hyatt, J. A. Cavity development in ice-rich permafrost, Pangnirtung, Baffin Island, Northwest Territories. *Permafr. Periglac. Process.* **3,** 293–313 (1992).

[126]Jolivel, M. & Allard, M. Thermokarst and export of sediment and organic carbon in the Sheldrake River watershed, Nunavik, Canada. *J. Geophys. Res. Earth Surf.* **118,** 1729–1745 (2013).

[127]Kokelj, S. V., Jenkins, R. E., Milburn, D., Burn, C. R. & Snow, N. The influence of thermokarst disturbance on the water quality of small upland lakes, Mackenzie Delta region, Northwest Territories, Canada. *Permafr. Periglac. Process.* **16,** 343–353 (2005).

[128]Kokelj, S. V. *et al.* Thawing of massive ground ice in mega slumps drives increases in stream sediment and solute flux across a range of watershed scales. *J. Geophys. Res. Earth Surf.* **118,** 681–692 (2013).

[129]Lacelle, D., Bjornson, J. & Lauriol, B. Climatic and geomorphic factors affecting contemporary (1950-2004) activity of retrogressive thaw slumps on the Aklavik plateau, Richardson Mountains, NWT, Canada. *Permafr. Periglac. Process.* **21,** 1–15 (2010).

[130]Lantuit, H. & Pollard, W. H. Fifty years of coastal erosion and retrogressive thaw slump activity on Herschel Island, southern Beaufort Sea, Yukon Territory, Canada. *Geomorphology* **95,** 84–102 (2008).

[131]Lantuit, H. *et al.* Modern and late Holocene retrogressive thaw slump activity on the Yukon coastal plain and Herschel Island, Yukon Territory, Canada. *Permafr. Periglac. Process.* **23,** 39–51 (2012).

[132]Lantz, T. C. & Kokelj, S. V. Increasing rates of retrogressive thaw slump activity in the Mackenzie Delta region, N.W.T., Canada. *Geophys. Res. Lett.* **35,** L06502 (2008).

[133]Lantz, T. C., Kokelj, S. V., Gergel, S. E. & Henry, G. H. R. Relative impacts of disturbance and temperature: Persistent changes in microenvironment and vegetation in retrogressive thaw slumps. *Glob. Chang. Biol.* **15,** 1664–1675 (2009).

[134]Lawson, D. E. Response of permafrost terrain to disturbance: A synthesis of observations from northern Alaska, U.S.A. *Arct. Alp. Res.* **18,** 1–17 (1986).

[135]Leibman, M. O. Cryogenic landslides on the Yamal Peninsula, Russia: Preliminary observations. *Permafr. Periglac. Process.* **6,** 259–264 (1995).

[136]Leibman, M., Gubarkov, A., Khomutov, A., Kizyakov, A. & Vanshtein, B. Coastal processes at the tabular-ground-ice-bearing area, Yugorsky Peninsula, Russia. in *Proc. 9th Int. Conf. Permafr.* (Kane, D. & Hinkel, K. M.) 1037–1042 (2008).

[137]Lewellen, R. I. *Permafrost erosion along the Beaufort Sea coast*. (1970).

[138]Lewkowicz, A. G. Nature and importance of thermokarst processes, Sand Hills Moraine, Banks Island, Canada. *Geogr. Ann. Ser. A, Phys. Geogr.* **69,** 321–327 (1987).

[139]Lewkowicz, A. G. Morphology, frequency and magnitude of active-layer detachment slides, Fosheim Peninsula, Ellesmere Island, N.W.T. in *Final Proceedings, 5th Can. Permafr. Conf.* 111–118 (1990).

[140]Lewkowicz, A. G. & Harris, C. Frequency and magnitude of active-layer detachment failures in discontinuous and continuous permafrost, northern Canada. *Permafr. Periglac. Process.* **16,** 115–130 (2005).

[141]Lewkowicz, A. G. & Harris, C. Morphology and geotechnique of active-layer detachment failures in discontinuous and continuous permafrost, northern Canada. *Geomorphology* **69,** 275–297 (2005).

[142]Lewkowicz, A. G. Dynamics of active-layer detachment failures, Fosheim Peninsula, Ellesmere. *Permafr. Periglac. Process.* **18,** 89–103 (2007).

[143]Liljedahl, A., Hinzman, L., Busey, R. & Yoshikawa, K. Physical short-term changes after a tussock tundra fire, Seward Peninsula, Alaska. *J. Geophys. Res. Earth Surf.* **112,** F02S07 (2007).

[144]Lipovsky, P. S. & Huscroft, C. in *Yukon Explor. Geol.* (Emond, D. S., Lewis, L. L. & Weston, L. H.) 181–195 (Yukon Geological Survey, 2006).

[145]Lyle, R. R., Hutchinson, D. J. & Preston, Y. in *Yukon Explor. Geol.* (Emond, D. S., Lewis, L. L. & Bradshaw, G. D.) 193–204 (Yukon Geological Survey, 2005).

[146]Mackay, J. R. Disturbances to the tundra and forest tundra environment of the western Arctic. *Can. Geotech. J.* **7,** 420–432 (1970).

[147]Malone, L., Lacelle, D., Kokelj, S. & Clark, I. D. Impacts of hillslope thaw slumps on the geochemistry of permafrost catchments (Stony Creek watershed, NWT, Canada). *Chem. Geol.* **356,** 38–49 (2013).

[148]McRoberts, E. C. & Morgenstern, N. R. The stability of thawing slopes. *Can. Geotech. J.* **11,** 447–469 (1974).

[149]Rudy, A. C. A., Lamoureux, S. F., Treitz, P. & Collingwood, A. Identifying permafrost slope disturbance using multi-temporal optical satellite images and change detection techniques. *Cold Reg. Sci. Technol.* **88,** 37–49 (2013).

[150]Seppälä, M. Piping causing thermokarst in permafrost, Ungava Peninsula, Quebec, Canada. *Geomorphology* **20,** 313–319 (1997).

[151]Sidorchuk, A. & Matveev, B. Channel process and erosion rates in the rivers of Yamal Peninsula in western Siberia. in *Proc. Canberra Symp.* 197–202 (1994).

[152]Singhroy, V., Murnaghan, K. & Couture, R. InSAR monitoring of a retrogressive thaw flow at Thunder River, lower Mackenzie. in *Proc. 6th Can. Permafr. Conf.* 1317–1322 (2010).

[153]Smirnov, V. M. Coastal processes along the west coast of Yamal. *Polar Geogr. Geol.* **10,** 317–321 (1986).

[154]Streletskiy, D. A., Shiklomanov, N. I., Nelson, F. E. & Klene, A. E. 13 years of observations at Alaskan CALM Sites: Long-term active layer and ground surface temperature trends. in *Proc. 9th Int. Conf. Permafr.* (Kane, D. & Hinkel, K. M.) 1–6 (2008).

[155]Toniolo, H., Kodial, P., Hinzman, L. D. & Yoshikawa, K. Spatio-temporal evolution of a thermokarst in Interior Alaska. *Cold Reg. Sci. Technol.* **56,** 39–49 (2009).

[156]Vogel, J., Schuur, E. A. G., Trucco, C. & Lee, H. Response of CO_2 exchange in a tussock tundra ecosystem to permafrost thaw and thermokarst development. *J. Geophys. Res. Biogeosciences* **114,** G04018 (2009).

[157]Wang, B., Paudel, B. & Li, H. Retrogression characteristics of landslides in fine-grained permafrost soils, Mackenzie Valley, Canada. *Landslides* **6,** 121–127 (2009).
